# Slow negative feedback enhances robustness of square-wave bursting

**DOI:** 10.1007/s10827-023-00846-y

**Published:** 2023-04-17

**Authors:** Sushmita Rose John, Bernd Krauskopf, Hinke M. Osinga, Jonathan E. Rubin

**Affiliations:** 1 Department of Mathematics, University of Pittsburgh, 301 Thackeray Hall, Pittsburgh, 15260 PA USA; 2 Department of Mathematics, University of Auckland, Private Bag 92019, Auckland, 1142 New Zealand

**Keywords:** Fast-slow decomposition, Bifurcation, Minimal models, Rhythms, Central pattern generators, Spikes

## Abstract

Square-wave bursting is an activity pattern common to a variety of neuronal and endocrine cell models that has been linked to central pattern generation for respiration and other physiological functions. Many of the reduced mathematical models that exhibit square-wave bursting yield transitions to an alternative pseudo-plateau bursting pattern with small parameter changes. This susceptibility to activity change could represent a problematic feature in settings where the release events triggered by spike production are necessary for function. In this work, we analyze how model bursting and other activity patterns vary with changes in a timescale associated with the conductance of a fast inward current. Specifically, using numerical simulations and dynamical systems methods, such as fast-slow decomposition and bifurcation and phase-plane analysis, we demonstrate and explain how the presence of a slow negative feedback associated with a gradual reduction of a fast inward current in these models helps to maintain the presence of spikes within the active phases of bursts. Therefore, although such a negative feedback is not necessary for burst production, we find that its presence generates a robustness that may be important for function.

**Supplementary Information:**

The online version contains supplementary material available at 10.1007/s10827-023-00846-y.

## Introduction

**Fig. 1:**
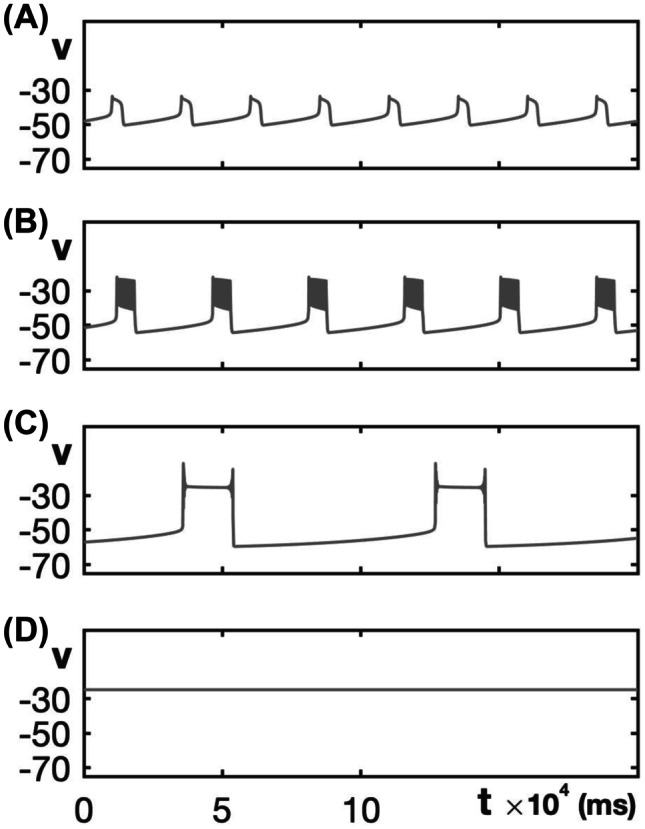
Non-spiking activity patterns. The voltages traces shown here are from the minimal Chay–Keizer model (7) and (8) with default parameter values. **A** Relaxation oscillations (RO) for $g_{ca}=1.2$. **B** Square-wave (SW) bursting for $g_{ca}=1.8$. **C** Pseudo-plateau (PP) bursting for $g_{ca}=3.2$. Note that although each active phase features an initial spike and a terminal spike, no other significant spiking occurs. **D** Depolarization block resulting from a stable critical point at elevated voltage for $g_{ca}=3.5$

In neuroscience, bursting refers to activity patterns in which a cell’s membrane potential alternates repeatedly between two phases: an active phase featuring a succession of spikes separated by relatively short inter-spike intervals and/or a sustained depolarization, and a silent or quiescent phase of little or no spiking. It has long been recognized that bursting patterns are closely connected with bifurcations in an underlying dynamical system (Rinzel, 1986). The original classification and analysis of bursting types relied on a fast-slow decomposition approach that falls within the realm of geometric singular perturbation theory (Dumortier and Roussarie, 2001; Jones, 1995; Wechselberger, 2020). Later work generalized the key idea of characterizing burst structure based on bifurcations associated with the transitions between active and silent phases (Izhikevich, 2000) and classifying bursting patterns in terms of unfoldings of higher-codimension bifurcation points (Bertram et al., 1995; Golubitsky et al., 2001; Krauskopf & Osinga, 2016; Osinga et al., 2012). In fact, these analyses embed bursting within a larger class of activity types that includes patterns such as relaxation oscillations (ROs; Fig. 1A), which also feature abrupt transitions between phases yet lack the spikes that occur during the active phases of bursts (Bertram & Rubin, 2017; Rinzel, 1986).

In this paper, we focus on two specific bursting activity patterns often observed in neural and endocrine cell recordings: square-wave (SW) and pseudo-plateau (PP) bursting (Fig. 1B, C), which are mathematically classified as fold-homoclinic and fold-sub-Hopf bursting, respectively (Izhikevich, 2000). These two bursting patterns stem from similar underlying bifurcation structures (Osinga et al., 2012; Tsaneva-Atanasova et al., 2010); however, in contrast to SW bursting, PP bursting does not produce reliable spiking activity, and often resembles an RO pattern (Fig. 1A, C).

Even though certain models are often referred to as models for one activity pattern or another, the same model can exhibit many different activity patterns, including multiple types of bursting, as parameters are varied, which the unfolding approach to burst analysis recognizes. Indeed, some minimal models for SW bursting yield a transition to PP (and vice versa) under small changes in parameter values (Osinga et al., 2012; Tabak et al., 2007; Teka et al., 2011a, b; Tsaneva-Atanasova et al., 2010). From a functional perspective, however, this effect may represent the emergence of a dysfunctional regime for a cell: the loss of spikes in the active phase associated with a transition from SW to PP bursting may result in a failure to release neurotransmitters or other signaling substances.

Since some cells are observed specifically to exhibit SW bursting, while others have been seen to produce both SW and PP patterns, we wondered if these differences could result from differences in the actual biophysical mechanisms expressed in these cells, rather than simply from observation of the dynamics within different parameter regimes. Indeed, spike production carries a significant energy cost (Shulman et al., 2004; Sokoloff, 1999), which suggests that when the firing of spikes is observed, this behavior is likely to be of functional importance and we might expect mechanisms to be present that enhance the robustness of spiking across parameter modulations. Similarly, some bursting cells feature fast inward sodium currents while others express fast inward calcium currents; although these are often considered interchangeable from a dynamics perspective (e.g., Izhikevich, 2007), which current is present may have implications for the robustness of bursting and spiking patterns that cells exhibit. The main motivation for this study is to understand what features promote the robustness of SW bursting – both to help explain the mechanisms that underlie differences in observed activity across neuron types and to guide the development of future models designed to capture such data.

In this work, we investigate the utility of a specific biophysical mechanism that we have recognized as enhancing the robustness of SW bursting in computational models. Bursting models feature a voltage-dependent fast inward current that helps to sustain the active phase, because it provides a fast positive feedback to the membrane potential (Izhikevich, 2007). We explore the effect on the robustness of SW bursting of adding a slow, voltage-dependent negative feedback associated to this inward current, which is a feature of fast sodium currents in neurons of certain types (Do & Bean, 2003; Milescu et al., 2010) and may also arise in fast calcium currents in some cases (Eckert & Chad, 1984; Zhang et al., 1994).

To carry out this analysis, we consider four classical, low-dimensional SW bursting models in their original forms, as well as with adjustments either to include a slow inactivation gate as part of the fast inward current, or to modify the kinetics of an already-present inactivation gate. This collection of models was selected to allow for consideration of fast inward sodium and calcium currents with a variety of mathematical formulations. We show that, over an appropriate range of the time constant for the respective inactivation gate, its inclusion broadens the range of maximal conductances $g_{ca}$ or $g_{na}$ of the fast inward current for which SW bursting — or a different form of spiking activity that can serve similar functional purposes in the context of a CPG (central pattern generator) circuit with inhibitory connections between populations (Bucher et al., 2015; Rubin & Smith, 2019) — occurs. We also show that, outside of this optimal range of inactivation timescales, SW bursting loses robustness, and the models easily transition from SW to PP bursting and other non-spiking patterns, including ROs and depolarization block (Fig. 1A, D), for which neurotransmitter release would be compromised.

The remainder of the paper is organized as follows. Section 2 starts with a brief introduction to geometric singular perturbation theory and discusses the bifurcation structure associated with SW and PP bursting patterns. We then introduce the four different bursting models in Section 2.1 and show how geometric singular perturbation theory is used to understand the various burst patterns exhibited by these models when $g_{ca}$ or $g_{na}$ is varied. In Section 2.2, we explain how we modify the models for our robustness analysis. The analysis of the robustness of SW bursting gained by including a slow, voltage-dependent negative feedback associated to the inward current follows in Section 3. The paper concludes with a discussion in Section 4.

## Preliminary analysis

In its simplest form, geometric singular perturbation theory assumes that a general model is defined in terms of an explicit fast-slow decomposition of the form

1 $$\begin{aligned} \left\{ \begin{array}{l} x^{\prime } = f(x, y, \epsilon ), \\ y^{\prime } = \epsilon \, g(x, y, \epsilon ), \end{array} \right. \end{aligned}$$

where $0 < \epsilon \ll 1$ is a small parameter, so that the fast variable is $x \in \mathbb {R}^m$ and the slow variable is $y \in \mathbb {R}^n$. In the limit $\epsilon \rightarrow 0$, system (1) reduces to a lower-dimensional, so-called fast subsystem

2 $$\begin{aligned} x' = f(x, y, 0), \end{aligned}$$

where the slow variable *y* plays the role of a constant parameter vector. The equilibria of the fast subsystem (2) form a manifold $\mathcal {C}$ in (*x*, *y*)-space,

$$\begin{aligned} \mathcal {C} = \{ (x, y) \in \mathbb {R}^m \times \mathbb {R}^n \; \mid \; f(x, y, 0) = 0 \}, \end{aligned}$$

which is called the critical manifold of system (1). We assume that $\mathcal {C}$ is Z-shaped with respect to the component of *x* that represents voltage *v*. This means that $\mathcal {C}$ has (at least) three co-existing equilibrium branches, parameterized by *y*. Ordered with respect to their corresponding *v*-components, we refer to these branches as the lower (silent) branch, the middle branch, and the upper (active) branch of $\mathcal {C}$.

For both SW and PP bursting, certain additional features must be present: firstly, that system (2) has a (lower) saddle-node bifurcation at some critical parameter value $y_\textsf{LSN}$, at which the lower and middle branches of $\mathcal {C}$ meet; and secondly, that system (2) has an Andronov–Hopf bifurcation along the upper branch of $\mathcal {C}$, which gives rise to a family $\mathcal {P}$ of periodic orbits of (2) parameterized by *y*. Note that this second requirement implies $m \ge 2$; that is, the fast variable *x* must be at least two dimensional. Crucially, this Andronov–Hopf bifurcation is subcritical in the PP case, which means that the orbits of $\mathcal {P}$ are unstable and, hence, system (2) does not produce stable spiking activity for initial conditions along the upper (active) branch of $\mathcal {C}$. For SW bursting, on the other hand, there exists a stable family of periodic orbits, together with a mechanism that induces a transition from the active phase to the silent phase. The originally described and most commonly considered form of SW bursting involves a supercritical Andronov–Hopf bifurcation for (2) on the upper (active) branch of $\mathcal {C}$ and a homoclinic bifurcation at which the family $\mathcal {P}$ of stable periodic orbits collides with a saddle equilibrium on the middle branch of $\mathcal {C}$ (Rinzel, 1986).

While the presence and order of specific bifurcations in the fast subsystem (2) help to predict the burst pattern exhibited by the full model, the burst pattern also depends on the relative location of the nullcline associated with the slow variable. In order for models to exhibit SW bursting, for example, it is necessary, although not sufficient, for the slow nullcline to intersect the middle branch of $\mathcal {C}$ at an equilibrium point below the homoclinic bifurcation; in particular, the full system must have a steady state that is of saddle type. We make sure this is the case over a sufficiently large range of parameters for all models considered in this paper.

### Models and parameter-dependence of bursting dynamics

As mentioned in the introduction, we select and study four different, low-dimensional SW bursting models with distinct formulations of the fast inward current. Each is presented in this section in its original form, and we discuss the parameter range for the maximal conductance of the fast inward current, $g_{ca}$ or $g_{na}$, over which SW bursting occurs.

#### Generic endocrine model

Tsaneva-Atanasova et al. (2010) introduced a generic endocrine model that exhibits both SW and PP bursting over physiologically relevant parameter ranges. The model is a system of differential equations for the membrane potential *v*, the gating variable *n* of the K$^+$ channel, and the calcium concentration *c* in the cytosol. The equations take the form

3 $$\left\{\begin{aligned} c_mv'&{}=-I_{Ca}(v)-I_K(v,n)-I_{K(Ca)}(v,c),\\ n'&{}=(n_\infty (v)-n)/\tau _n,\\ c'&{}=-f_c(\alpha I_{Ca}(v)+k_pc)\end{aligned}\right.$$

for constants $c_m$, $\tau _n$, $f_c$, $\alpha$, and $k_p$. The expressions for the currents and steady state activation functions are given by:

4 $$\begin{aligned} I_{Ca}(v) &{}= g_{ca} \, m_{\infty }^2(v) \, (v - e_{ca}), \\ I_K(v,n) &{}= g_{k} \, n \, (v - e_{k}),\\ I_{K(Ca)}(v,c) &{}= g_{kca} \, \left( \dfrac{c^4}{c^4+k_s^4} \right) \, (v - e_{k}), \\ m_{\infty }(v) &{}= (1 + e^{(v_m-v)/s_m})^{-1}, \\ n_{\infty }(v) &{}= (1 + e^{(v_n-v)/s_n})^{-1}. \end{aligned}$$

**Fig. 2:**
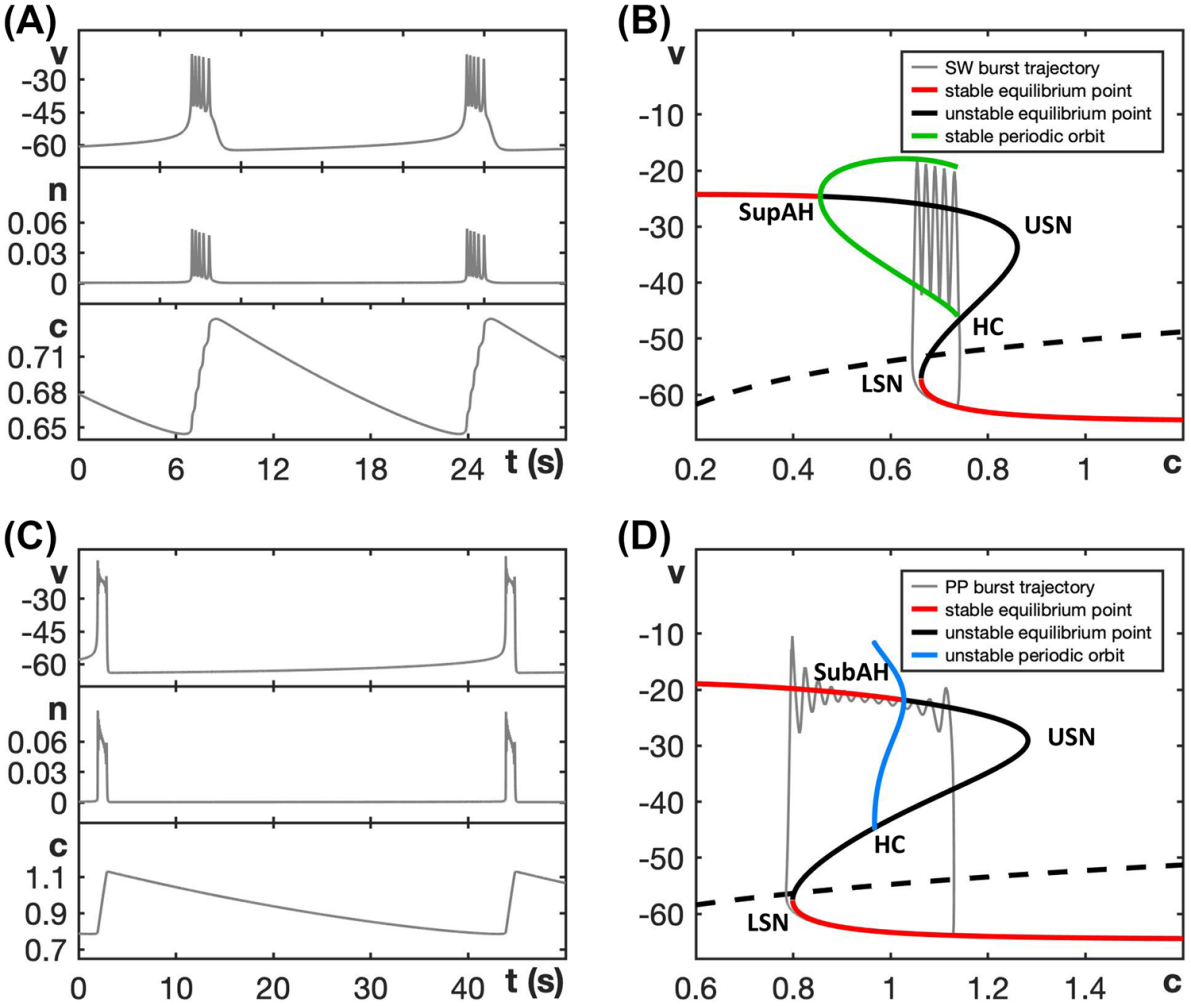
Fast-slow decomposition for the generic endocrine model (3) and (4). **A** SW bursting for default parameter values given in Table 1. **B** Bifurcation diagram of the model’s fast subsystem with respect to the slow variable *c*, with bifurcation points labeled and the burst trajectory, which evolves clockwise, overlaid in gray. Oscillations start after the trajectory jumps up from the lower left saddle node (LSN) and stop when it reaches the homoclinic (HC). **C** The model exhibits PP bursting when $g_{ca}$ is increased to 1.5; note that the ranges of *c* in (**A**) and (**C**) are different. **D** Bifurcation diagram as in (**C**) but with $g_{ca} = 1.5$; the PP burst trajectory, which also evolves clockwise, is again overlaid in gray. The labels SupAH (**B**) and SubAH (**D**) refer to supercritical and subcritical Andronov–Hopf bifurcations, respectively

We choose default parameter values as given in Table 1, for which the model exhibits the SW bursting pattern shown in Fig. 2A. Indeed, non-dimensionalization (see the Appendix) shows that the three-dimensional system (3) and (4) readily separates into fast and slow equations, because *v* changes at a rate $R_v \approx 716$ that is faster than the rate $R_n \approx 33$ for *n* which, in turn, is significantly faster than the rate $R_c \approx 1.7$ for *c*. We consider *v* and *n* as two fast variables and *c* as one slow variable, so that system (3) and (4) has the lowest possible dimensions for SW bursting; the alternative pairing of one fast and two slow variables would be relevant for studying canard dynamics (Vo et al., 2013), but we do not consider this here. More details about the model can also be found in (Tsaneva-Atanasova et al., 2010).

**Table 1:** Generic Endocrine Model (3) and (4): default parameter values

$c_m$	0.00314159 nF	$g_{ca}$	0.81 nS
$g_k$	2.25 nS	$g_{kca}$	0.2 nS
$e_k$	$-65$ mV	$e_{ca}$	0 mV
$v_m$	$-22.5$ mV	$v_n$	0 mV
$s_m$	12 mV	$s_n$	8 mV
$\tau _n$	0.03 s	$k_s$	1.25 $\mu$M
$f_c$	0.003	$k_{p}$	5 s$^{-1}$
$\alpha$	14 $\mu M/pC$		

The fast subsystem, consisting of the (*v*, *n*)-equations in (3) and (4), and its attractors can be studied by considering the slow variable *c* as a bifurcation parameter. The corresponding bifurcation diagram, shown in Fig. 2B, forms a scaffold for understanding the burst pattern that the full model produces. Specifically, based on this fast-slow decomposition, we can assume that any general initial position with slow variable $c = c_0$ lies on a trajectory that predominantly evolves under the fast dynamics to one of the attractors that exists in the fast subsystem for $c = c_0$. Subsequently, the sign of $c'$ will determine whether the trajectory drifts to the left or right along the corresponding attractor branch until either this branch terminates and a transition to a new attractor occurs, the trajectory goes off to infinity, or a stable state for the full system is reached. In Fig. 2B, for (3) and (4) with default parameter values, the *c*-nullcline (dashed curve) cuts through the middle branch of the critical manifold $\mathcal {C}$, just below HC in the bifurcation diagram. According to the equation for *c* in (3) and (4), we have $c' < 0$ below this nullcline. Hence, *c* is decreasing during the silent phase and, as suggested by the bifurcation diagram of the fast subsystem, the active phase of the SW burst starts due to a jump up in potential *v* from the *c*-value at which the fast subsystem undergoes a saddle-node bifurcation (LSN). For this *c*-value, the attractor of the fast subsystem at elevated voltage is a periodic orbit, part of a family of such orbits that originates in a supercritical Andronov–Hopf bifurcation (SupAH). Thus, oscillations result, and they continue as *c* increases, according to its equation in (3) and (4), until a homoclinic bifurcation (HC) occurs. At that bifurcation, the trajectory returns to the silent phase, where it is attracted to the stable equilibria on the lower (silent) branch of $\mathcal {C}$.

When $g_{ca}$ is increased from its default value of 0.81 to $g_{ca} = 1.5$, the model exhibits the qualitatively different PP bursting pattern (Fig. 2C). The bifurcation diagram of the fast subsystem with respect to the variable *c* has changed correspondingly (Fig. 2D). In particular, we see that the Andronov–Hopf bifurcation point has now moved to a larger *c*-value and has changed criticality to become subcritical (SubAH). Therefore, the fast subsystem now has a family of unstable periodic orbits. Hence, after the jump up from LSN, the trajectory is attracted to the upper branch of the critical manifold $\mathcal {C}$, which comprises stable equilibria of the fast subsystem. Since these equilibria are foci, the trajectory spirals around the upper branch of $\mathcal {C}$ while slowly moving to the right with respect to *c*. This behavior generates a voltage plateau in the active phase, accompanied by rapidly decaying oscillations in lieu of spikes (Fig. 2C). The upper branch of $\mathcal {C}$ loses stability at SubAH, and after a small delay associated with the slow passage through an Andronov–Hopf bifurcation (Baer et al., 1989; Baer & Gaekel, 2008; Neishtadt, 1987, 1988), the trajectory jumps down to the silent phase where it flows back to LSN to complete a burst cycle.

**Fig. 3:**
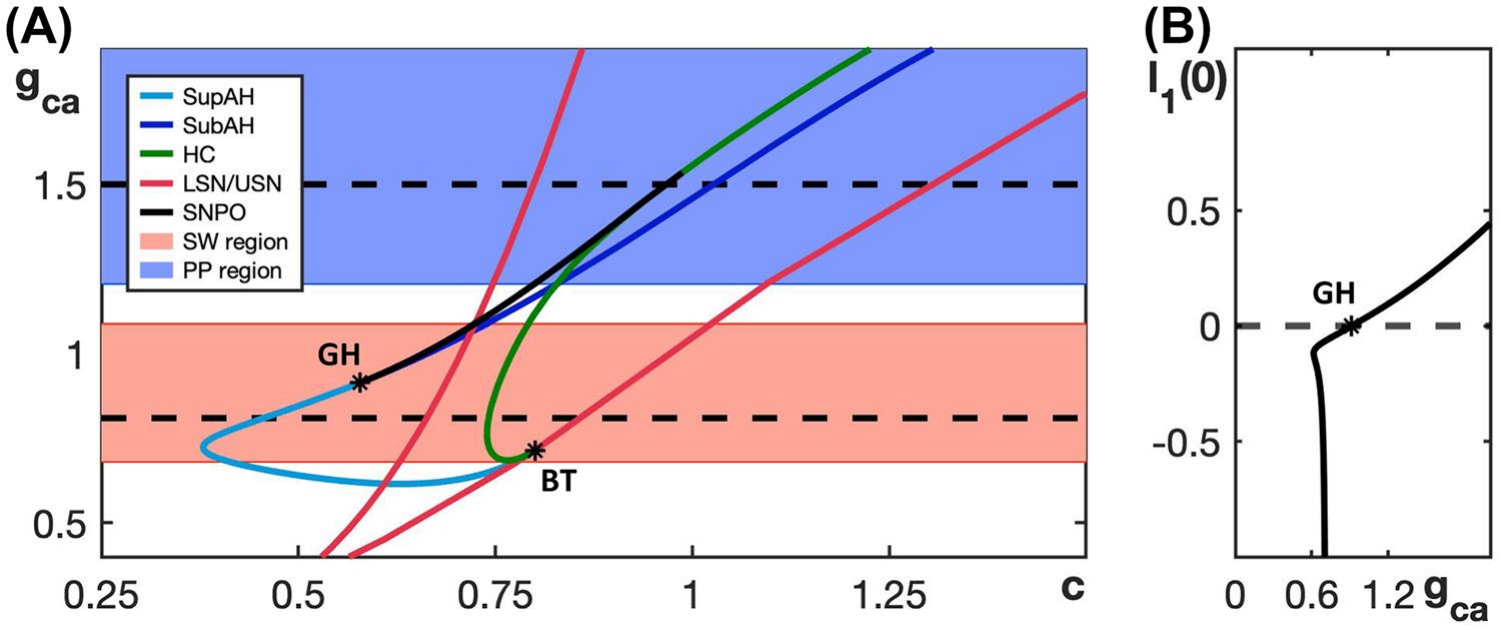
Dependence on $g_{ca}$ of bifurcation curves for the fast subsystem of the generic endocrine model (3) and (4). **A** Two-parameter bifurcation diagram in the $(c, g_{ca})$-plane. The locus AH of Andronov–Hopf bifurcations (blue) comprises the two curves SupAH and SubAH that meet at the generalized Hopf point labeled GH (left black star); SupAH and the curve HC of homoclinic bifurcations merge and end at a Bogdanov–Takens point (BT; right black star) on the curve USN of saddle-node bifurcations (red). The SW and PP bursting regions are shaded red and blue, respectively. The black dashed lines correspond to the examples of SW bursting for $g_{ca}=0.81$ and PP bursting for $g_{ca}=1.5$ shown in Fig. 2. **B** Lyapunov coefficent along the curve AH. The Andronov–Hopf bifurcation is supercritical until this coefficient increases through 0 for $g_{ca}$ just below 1, corresponding to the point GH, and subcritical for $g_{ca}$-values above that

The bifurcation diagrams in Fig. 2 display SW and PP bursting patterns produced by the generic endocrine model (3) and (4) for two fixed values of $g_{ca}$. The robustness of these patterns and the transition between them can be studied more systematically by considering a two-parameter bifurcation diagram. Specifically, we can follow the codimension-one bifurcations labeled LSN, USN, SupAH, SubAH and HC in Fig. 2B, D as curves in the two-parameter $(c, g_{ca})$-plane. The resulting bifurcation diagram is displayed in Fig. 3A.

The two-parameter bifurcation diagram shows how the bifurcation points change, and in some cases meet and disappear, when $g_{ca}$ varies away from its default value of 0.81 (bottom dashed line). In particular, the curves SupAH (light blue) and HC (green) of supercritical Andronov–Hopf and homoclinc bifurcations, respectively, end on the curve USN of saddle-node bifucation (red) at the codimension-two Bogdanov–Takens point BT (right black star). Furthermore, the curve SupAH transitions to SubAH by changing criticality at the generalized Hopf point GH (black star just below $g_{ca} = 1$ on the curve AH in the diagram), which occurs when the first Lyapunov coefficient associated with the Andronov–Hopf bifurcation changes sign (Fig. 3B); this first Lyapunov coefficient was computed numerically with MATCONT (Dhooge et al., 2003). The curve subAH (dark blue) of subcritical Andronov–Hopf bifurcations then moves into the V-shaped region between the two curves LSN and USN. At the point GH, a curve of saddle-node bifurcation of periodic orbits (SNPO) originates and progresses to larger *c*-values as $g_{ca}$ continues to increase, until it ends just above $g_{ca} = 1.5$ on the curve HC. In the remainder of the paper, we will denote as AH the locus or curve of Andronov-Hopf bifurcation comprised of the components SupAH and SubAH.

The lower black dashed line in Fig. 3A corresponds to the bifurcation diagram for $g_{ca} = 0.81$ in Fig. 2B that gives rise to SW bursting. In the direction of increasing *c*, we successively encounter the supercritical Andronov–Hopf bifurcation SupAH (light blue), the saddle-node bifurcation LSN (red), the homoclinic bifurcation HC (green), and the other saddle-node bifurcation USN (red). If we use $c_\textsf{X}$ to denote the *c*-value at which a bifurcation of type $\textsf{X}$ occurs, then the order of bifurcations for fixed $g_{ca} = 0.81$ is $c_\textsf{SupAH}< c_\textsf{LSN}< c_\textsf{HC}< c_\textsf{USN}$. This order of bifurcations is maintained for lower values of $g_{ca}$, until HC disappears, just below the point BT. Hence, since $c_\textsf{SupAH} < c_\textsf{LSN}$, the active phase is characterized by stable periodic orbits, and persists until $c \approx c_\textsf{HC}$; we conclude that these $g_{ca}$-values all give rise to SW bursting. Similarly, for larger $g_{ca}$-values, even though SubAH and LSN cross, the change in criticality at GH implies that the active phase is still characterized by stable periodic orbits until the saddle-node bifurcation of periodic orbits SNPO occurs after LSN; that is, for SW bursting, we require $c_\textsf{SNPO} < c_\textsf{LSN}$.

The order of the bifurcations along the black dashed line for $g_{ca} = 1.5$ in Fig. 3A, which corresponds to PP bursting shown in Fig. 2D, is $c_\textsf{LSN}< c_\textsf{SNPO}< c_\textsf{HC}< c_\textsf{SubAH} < c_\textsf{USN}$; it is important that $c_\textsf{SNPO}$ is only just smaller than $c_\textsf{HC}$, which means that this order generates a PP pattern that is qualitatively similar to that for $g_{ca}$-values above the point where SNPO ends, which feature the bifurcation sequence $c_\textsf{LSN}< c_\textsf{HC}< c_\textsf{SubAH} < c_\textsf{USN}$. While we did not check all $g_{ca}$-values, this order of bifurcations is maintained until at least $g_{ca} = 2$.

The red and blue shaded regions in Fig. 3A show the ranges of $g_{ca}$-values over which the generic endocrine model (3) and (4) can potentially exhibit SW and PP bursting, respectively. Choosing parameters in one of these regions is, in fact, not sufficient to ensure that the corresponding burst pattern occurs, since the actual burst pattern also depends on the position of the *c*-nullcline — which changes with $g_{ca}$ due to the presence of $I_{Ca}$ in the *c*-equation in (3) and (4) — and the speed at which *c* evolves. We conclude from this diagram, however, that SW bursting can at most be maintained for $0.65< g_{ca} < 1.1$.

**Fig. 4:**
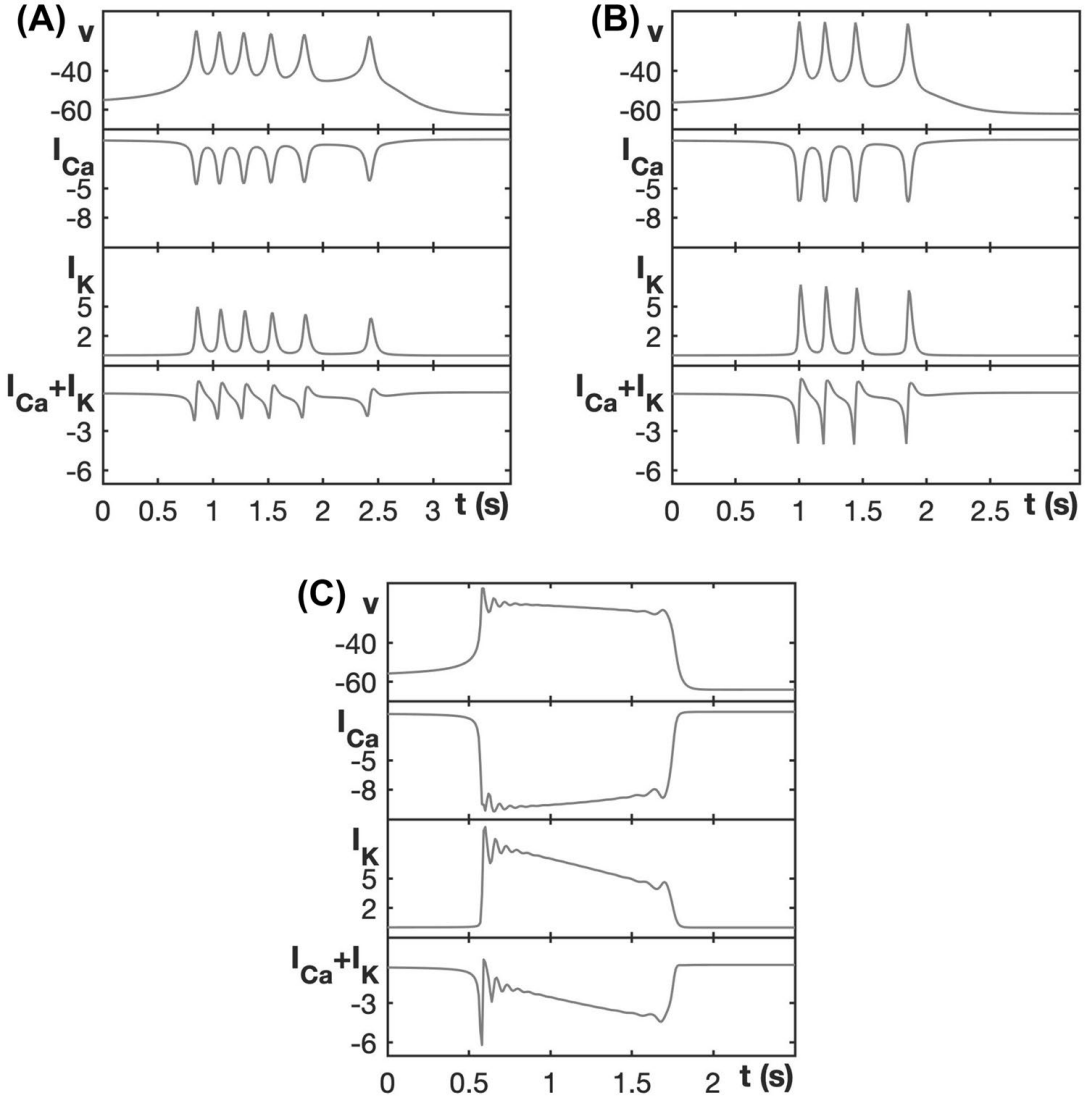
Burst patterns exhibited by the generic endocrine model (3) and (4) for different values of $g_{ca}$, along with associated currents. **A** SW bursting at $g_{ca}=0.75$. **B** SW bursting at $g_{ca}=1.0$ with larger amplitude spikes than in (**A**). **C** PP bursting for $g_{ca} = 1.6$

Figure 4 compares the burst patterns of the generic endocrine model (3) and (4) for different values of $g_{ca}$. At $g_{ca} = 0.75$, the model exhibits SW bursting (Fig. 4A) that is very similar to that for the default value $g_{ca} = 0.81$ (Fig. 2A). When $g_{ca}$ is increased to 1.0, the model still exhibits SW bursting (Fig. 4B), but the increase in $g_{ca}$ strengthens $I_{Ca}$, which results in more elevated *v* values at peaks of the bursts. At this elevated *v*, the current $I_K$ activates more strongly compared to the previous case, resulting in stronger hyperpolarizations between spikes and fewer spikes in the burst. When $g_{ca}$ is increased still further to 1.6, the activity pattern transitions to PP bursting (Fig. 4C). This case yields the strongest $I_{Ca}$ activation of the three; indeed, despite the induced elevation of *v* and corresponding strong activation of $I_K$, the latter current cannot overcome $I_{Ca}$ and cause repolarization. Thus, the equilibria on the upper branch of the critical manifold $\mathcal {C}$ stabilize and spike oscillations during the active phase are prevented.

#### Sodium-potassium minimal model

The sodium-potassium minimal model introduced in (Izhikevich, 2007) is an example of an SW burster comprised of only the basic essentials needed to burst. This model consists of the following differential equations:

5 $$\left\{ \begin{aligned} c_m \, v' & = -I_L(v) - I_{Na}(v) - I_K(v,n) - I_{S}(v,s) + I, \\ n' &{}= (n_{\infty }(v) - n)/\tau _n, \\ s' &{}= (s_{\infty }(v) - s)/\tau _s. \end{aligned} \right.$$

The expressions for the currents and steady state activation functions for the model are given by:

6 $$\begin{aligned} I_{L}(v) &{}= g_l \, (v - e_l), \\ I_{Na}(v) &{}= g_{na} \, m_{\infty }(v) \, (v - e_{na}), \\ I_K(v,n) &{}= g_{k} \, n \, (v - e_{k}), \\ I_{S}(v,s) &{}= g_{km} \, s \, (v - e_{k}), \\ m_{\infty }(v) &{}= (1 + e^{(v_m-v)/s_m})^{-1}, \\ n_{\infty }(v) &{}= (1 + e^{(v_n-v)/s_n})^{-1}, \\ s_{\infty }(v) &{}= (1 + e^{(v_s-v)/s_s})^{-1}. \end{aligned}$$

**Fig. 5:**
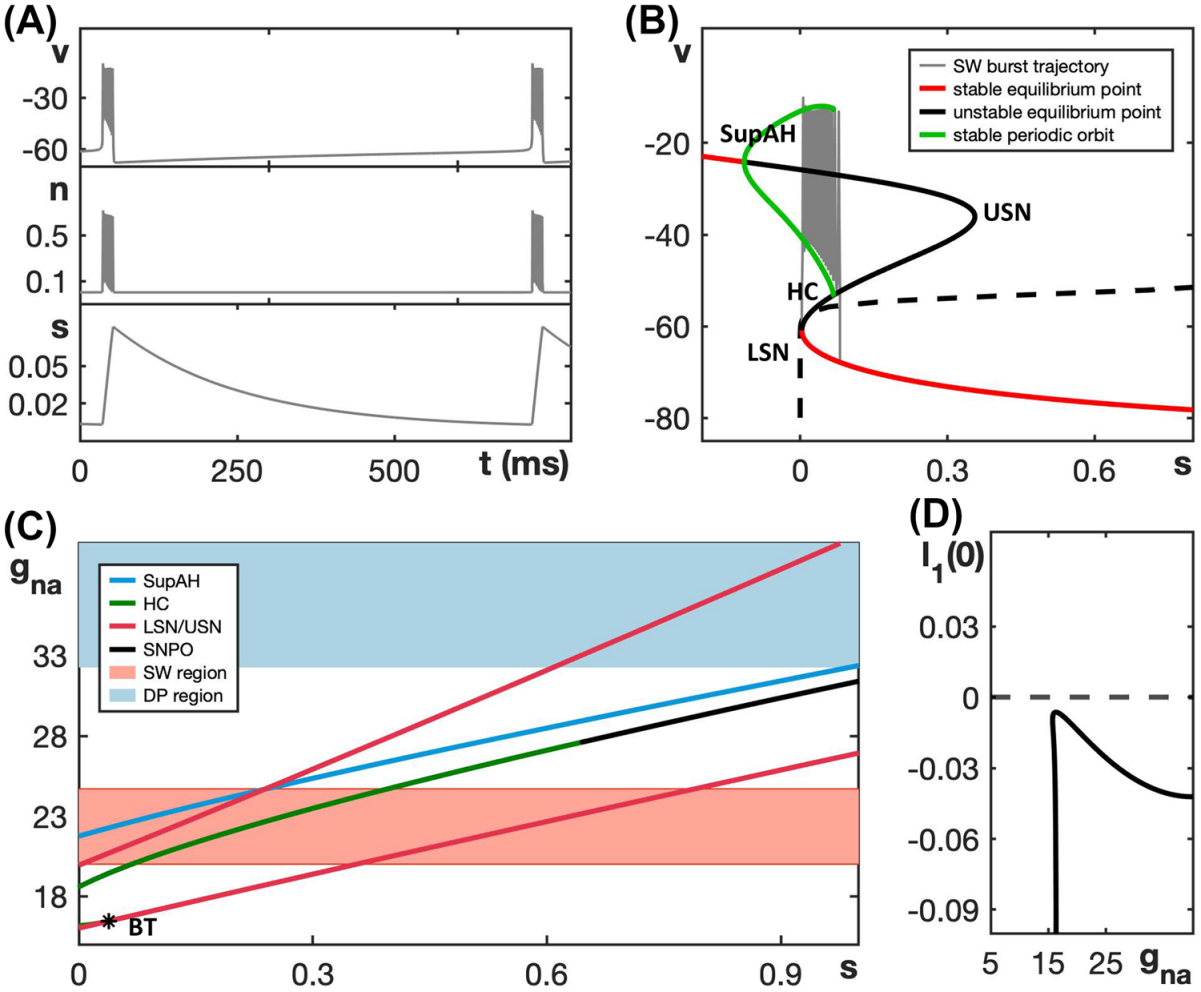
Dynamics and bifurcation structure for the sodium-potassium minimal model (5) and (6). **A** SW bursting for the default parameters given in Table 2. **B** Bifurcation diagram of the model’s fast subsystem with respect to the slow variable *s* for default parameter values, with the SW burst overlaid in gray. **C** Two-parameter bifurcation diagram of the fast subsystem in the $(s, g_{na})$-plane; colors are as in Fig. 3A and the SW bursting region is shaded red. For realistic values ($s<1$), this model does not transition to PP. The light-blue shaded region corresponds to $g_{na}$-values for which the full system has a stable steady state at elevated *v*. **D** Lyapunov coefficient along the curve SupAH. The Andronov–Hopf bifurcation is supercritical until around $g_{na}=65$, which lies outside the range shown in panel (**C**)

Note that $I_S$ denotes a potassium current with gating that evolves much slower than that for $I_K$. Again, we choose default parameter values, given in Table 2, for which the model exhibits SW bursting as shown in Fig. 5A. The bifurcation diagram of the model’s fast subsystem for default parameter values is shown in Fig. 5B. Notice that the order of bifurcations, in the direction of increasing *s*, is the same as in Fig. 2B, that is, $s_\textsf{SupAH}< s_\textsf{LSN}< s_\textsf{HC}< s_\textsf{USN}$.

**Table 2:** Sodium-Potassium Minimal Model (5)–(6): default parameter values

$c_m$	1 pF	*I*	5 pA
$g_l$	8 nS	$g_{na}$	20 nS
$g_k$	9 nS	$g_s$	5 nS
$e_l$	$-80$ mV	$e_{na}$	60 mV
$e_k$	$-90$ mV	$v_m$	$-20$ mV
$v_n$	$-25$ mV	$v_s$	$-20$ mV
$s_m$	15 mV	$s_n$	5 mV
$s_s$	5 mV	$\tau _n$	0.15 ms
$\tau _s$	200 ms		

By comparing timescales after non-dimensionalization of this model (see the Appendix), we find that the timescale constants of *v*, *n* and *s* are approximately $R_v \approx 20$, $R_n \approx 6.57$ and $R_s \approx 0.005$, respectively. The rate $R_v \approx 20$ varies linearly with $g_{na}$ as long as $g_{na} > 9 = g_k$; if $g_{na}$ is decreased below this value, $R_v \approx 9$ is determined by $g_k$ instead and any further decrease in $g_{na}$ would not affect $R_v$. Hence, the variables *v* and *n* are considerably faster than *s*, irrespective of the value for $g_{na}$.

Even though the sodium-potassium minimal model (5) and (6) is designed to exhibit SW bursting, it is capable of other activity patterns. For example, Supplemental Fig. 1 shows the non-spiking pattern generated for $g_{na} = 35$ in which all solutions are attracted to a stable steady state at an elevated voltage level, which corresponds to a state of depolarization block. For this large value of $g_{na}$, the nullcline of the slow variable *s* intersects the upper branch of the critical manifold, which gives rise to a stable steady state of the full system. However, SW bursting is already lost for smaller $g_{na}$-values. Figure 5C shows the two-parameter bifurcation diagram of the fast subsystem in the $(s, g_{na})$-plane. As in Section 2.1.1, the ordering of bifurcation curves suggests that system (5) and (6) can potentially exhibit SW bursting for $g_{na}$-values between 20 and 25, if the slow dynamics is tuned appropriately; this region is again shaded red. We computed the first Lyapunov coefficient associated with the Andronov-Hopf bifurcation (Fig. 5D) and found that it is negative for the default $g_{na}$ and remains so up until a much larger value, $g_{na} \approx 65$. Hence, this system does not transition to PP bursting, at least not for $s \in (0,1)$, the physically relevant range. Instead, for $g_{na} > 32$ or so, system (5) and (6) moves into a state of depolarization block, which is the region shaded light blue in Fig. 5B.

#### Minimal Chay-Keizer model

Next, we consider the minimal Chay–Keizer model described in (Rinzel, 1986; Rinzel & Lee, 1986). This model takes the form:

7 $$\begin{aligned} \left\{ \begin{aligned} c_m \, v^{\prime }&= -I_{L}(v) - I_{Ca}(v) -I_K(v, n) - I_{K(Ca)}(v, c), \\ n^{\prime }&= (n_{\infty }(v) - n)/\tau _n(v), \\ c^{\prime }&= -f_c \, (\alpha \, I_{ca}(v) \, + k_{p} \, c). \end{aligned} \right. \end{aligned}$$

The currents and steady state activation functions for the model are given by:

8 $$\begin{aligned} I_L(v) &{}= g_l \, (v - e_l), \\ I_{Ca}(v) &{}= g_{ca} \, m_{\infty }^3(v) \, h_{\infty }(v) \, (v - e_{ca}), \\ I_K(v, n) &{}= g_{k} \, n \, (v - e_{k}), \\ I_{K(Ca)}(v, c) &{}= g_{kca}\, {\displaystyle \frac{c}{1+c}} \, (v - e_{k}), \\ a_m(v) &{}= {\displaystyle \frac{0.1 \, (v + 25)}{1 - e^{-0.1(v+25)}}}, \\ b_m(v) &{}= 4 \, e^{-(v + 50)/18}, \\ m_{\infty }(v) &{}= {\displaystyle \frac{a_m(v)}{a_m(v) + b_m(v)}}, \\ a_n(v) &{}= {\displaystyle \frac{0.01 \, (v + 20)}{1 - e^{-0.1(v+20)}}}, \\ b_n(v) &{}= 0.125 \, e^{-(v+30)/80}, \\ n_{\infty }(v)&{}= {\displaystyle \frac{a_n(v)}{a_n(v) + b_n(v)}}, \\ \tau _n(v) &{}= {\displaystyle \frac{3.33}{a_n(v) + b_n(v)}}, \\ a_h(v) &{}= 0.07 \, e^{-(v+50)/20}, \\ b_h(v) &{}= {\displaystyle \frac{1}{e^{-0.1(v+20)} + 1}}, \\ h_{\infty }(v) &{}= {\displaystyle \frac{a_h(v)}{a_h(v) + b_h(v)}}. \end{aligned}$$

**Fig. 6:**
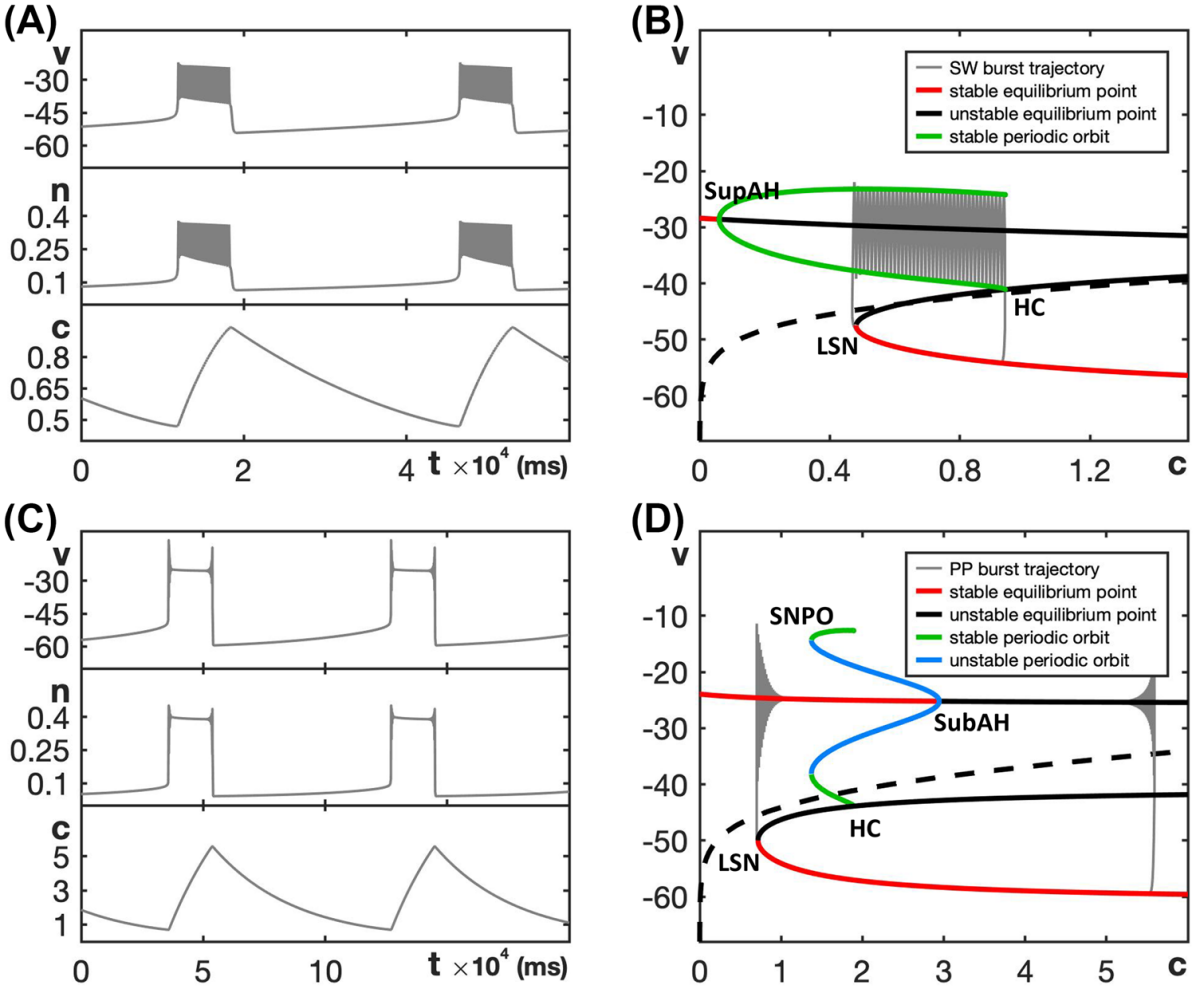
Dynamics and bifurcation structure for the minimal Chay–Keizer model (7) and (8). **A** SW bursting for the default parameters given in Table 3. **B** Bifurcation diagram of the model’s fast subsystem with respect to the slow variable c for the default value of  $g_{ca}$. The SW burst trajectory is overlaid in gray. **C** The model exhibits PP bursting for $g_{ca}=3.2$; note the difference in *c*-range between (**A**) and (**C**). **D** The bifurcation diagram as in (**B**) but with $g_{ca} = 3.2$

We choose the default parameter values given in Table 3, for which the model exhibits SW bursting as displayed in Fig. 6A.

**Table 3:** Minimal Chay–Keizer Model (7) and (8): default parameter values

$c_m$	1 $\mu$F/$cm^2$	$g_l$	0.006985 mS/$cm^2$
$g_{ca}$	1.79934 mS/$cm^2$	$g_k$	1.69765 mS/$cm^2$
$g_{kca}$	0.0104998 mS/$cm^2$	$e_k$	$-75$ mV
$e_{ca}$	100 mV	$e_l$	$-40$ mV
$k_{p}$	0.00513 ms$^{-1}$	$f_c$	0.0058
$\alpha$	0.02591 $\mu M/nC$		

Non-dimensionalization (see the Appendix) shows that the timescale constants of *v* and *c* in this model are $R_v \approx 1.8$, $R_{c}\approx 0.004$ respectively, while the time constant $R_n$ for *n* depends on *v* and varies between 0.05 to 0.1 over the relevant range of *v* values. In this model, both $R_v$ and $R_{c}$ depend on $g_{ca}$. We choose an upper bound of 4 on $g_{ca}$, which is double the default value. At this maximal value, we have $R_v \approx 4$ and $R_{c} \approx 0.008$, or roughly twice the default values. Even with these timescale constants, *v* and *n* can be considered as fast compared to *c*.

**Fig. 7:**
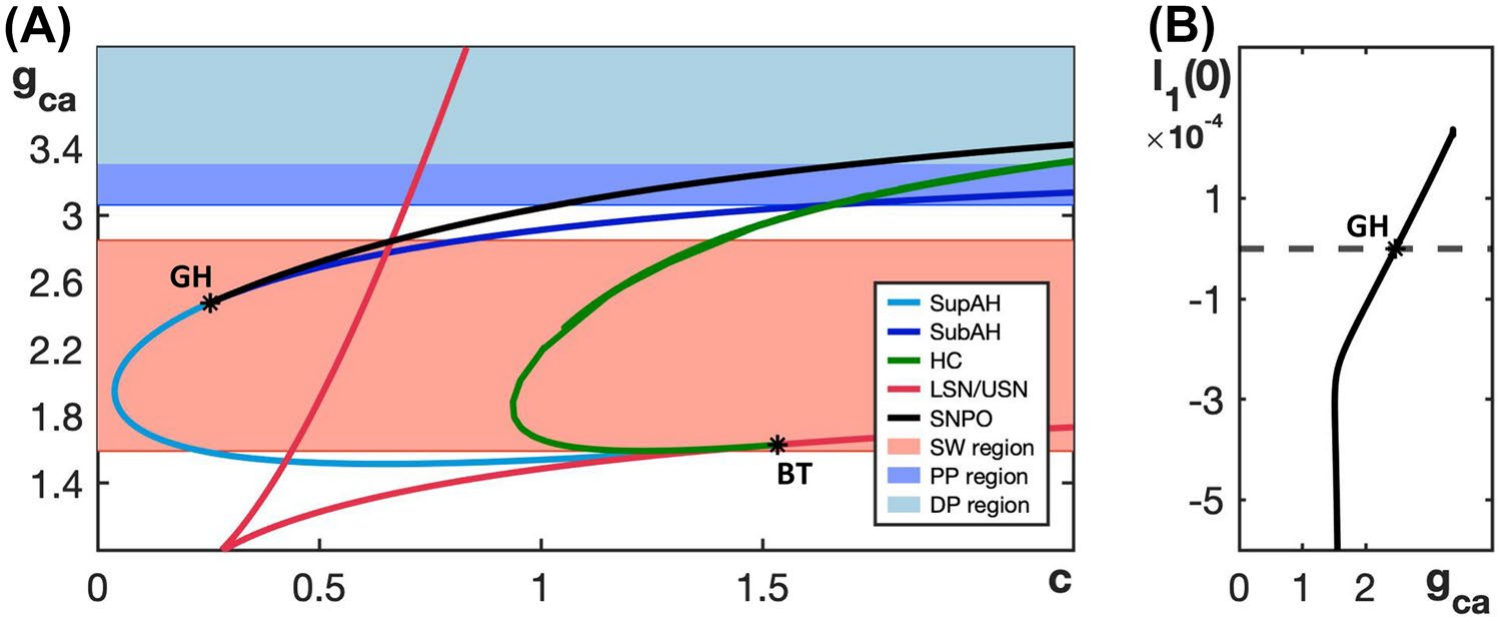
**A** Two-parameter bifurcation diagram of the fast subsystem of the minimal Chay–Keizer model (7) and (8) in the $(c, g_{ca})$-plane; colors are as in Fig. 3A and the SW bursting region is shaded red, the narrow PP region is shaded blue, and the light-blue shaded region just above that corresponds to a state of depolarization block. **B** Lyapunov coefficient along the curve AH, comprised of SupAH and SubAH. The Andronov–Hopf bifurcation is supercritical for $g_{ca}$ below the axis crossing close to $g_{ca}=2.5$ and subcritical for larger $g_{ca}$. Note that the curve ends at a  $g_{ca}$-asymptote

The minimal Chay–Keizer model (7) and (8) exhibits SW bursting for the default parameter values given in Table 3 and PP busting when $g_{ca}$ increases to 3.2; see Fig. 6A, C. The corresponding bifurcation diagrams of the model’s fast subsystem with respect to the slow variable are shown in Fig. 6B, D. The two-parameter bifurcation diagram in the $(c, g_{ca})$-plane shown in Fig. 7A illustrates how the bifurcations of the fast subsystem depend on $g_{ca}$. Based on the relative order of the bifurcation curves, we conclude that the minimal Chay–Keizer model (7) and (8) can potentially exhibit SW bursting for $1.5<g_{ca} <2.8$ (red shaded region) and PP bursting for $g_{ca}$ near 3.2 (narrow blue shaded region). When $g_{ca}$ is increased further, the Andronov–Hopf bifurcation moves to larger values of *c*, such that the nullcline of the slow variable *c* intersects the upper branch of the critical manifold at a stable equilibrium point. In doing so, the full system now has a stable steady state and hence, for sufficiently large $g_{ca}$-values, the system exhibits depolarization block with voltage suspended at an elevated level (light-blue shaded region).

#### Butera model

The Butera model is a seminal minimal model used to study rhythm generation in respiratory neurons (Butera et al., 1999). This model consists of the following differential equations:

9 $$\left\{ \begin{aligned} c_m \, v' &{}= -I_L(v) - I_{Na}(v,n) - I_K(v,n) \\ & \quad\, -I_{NaP}(v,p) - I_{ton}(v), \\ n' &{}= (n_{\infty }(v) - n)/\tau _n, \\ p' &{}= (p_{\infty }(v) - p)/\tau _p(v), \end{aligned} \right.$$

where $I_{NaP}$ denotes a persistent sodium current. The expressions for the currents and steady state activation functions are as follows:

10 $$\begin{aligned} I_L(v) &{}= g_{l} \, (v - e_l), \\ I_{Na}(v, n) &{}= g_{na} \, m_{\infty }^3(v) \, (1 - n) \, (v - e_{na}), \\ I_K(v,n) &{}= g_{k} \, n^4 \, (v - e_{k}), \\ I_{NaP}(v, p) &{}= g_{nap} \, m \, p_{\infty }(v) \, p \, (v - e_{na}), \\ I_{ton}(v) &{}= g_{ton} \, (v - e_{syn}), \\ m_{\infty }(v) &{}= (1 + e^{(v_m-v)/s_m})^{-1}, \\ n_{\infty }(v) &{}= (1 + e^{(v_n-v)/s_n})^{-1}, \\ mp_{\infty }(v) &{}= (1 + e^{(v_{mp}-v)/s_{mp}})^{-1}, \\ p_{\infty }(v) &{}= (1 + e^{(v_p-v)/s_p})^{-1}, \\ \tau _n(v) &{}= \tau _n \, (\cosh ((v - v_n)/(2s_n)))^{-1}, \\ \tau _p(v) &{}= \tau _p \, (\cosh ((v - v_p)/(2s_p)))^{-1}. \end{aligned}$$

**Fig. 8:**
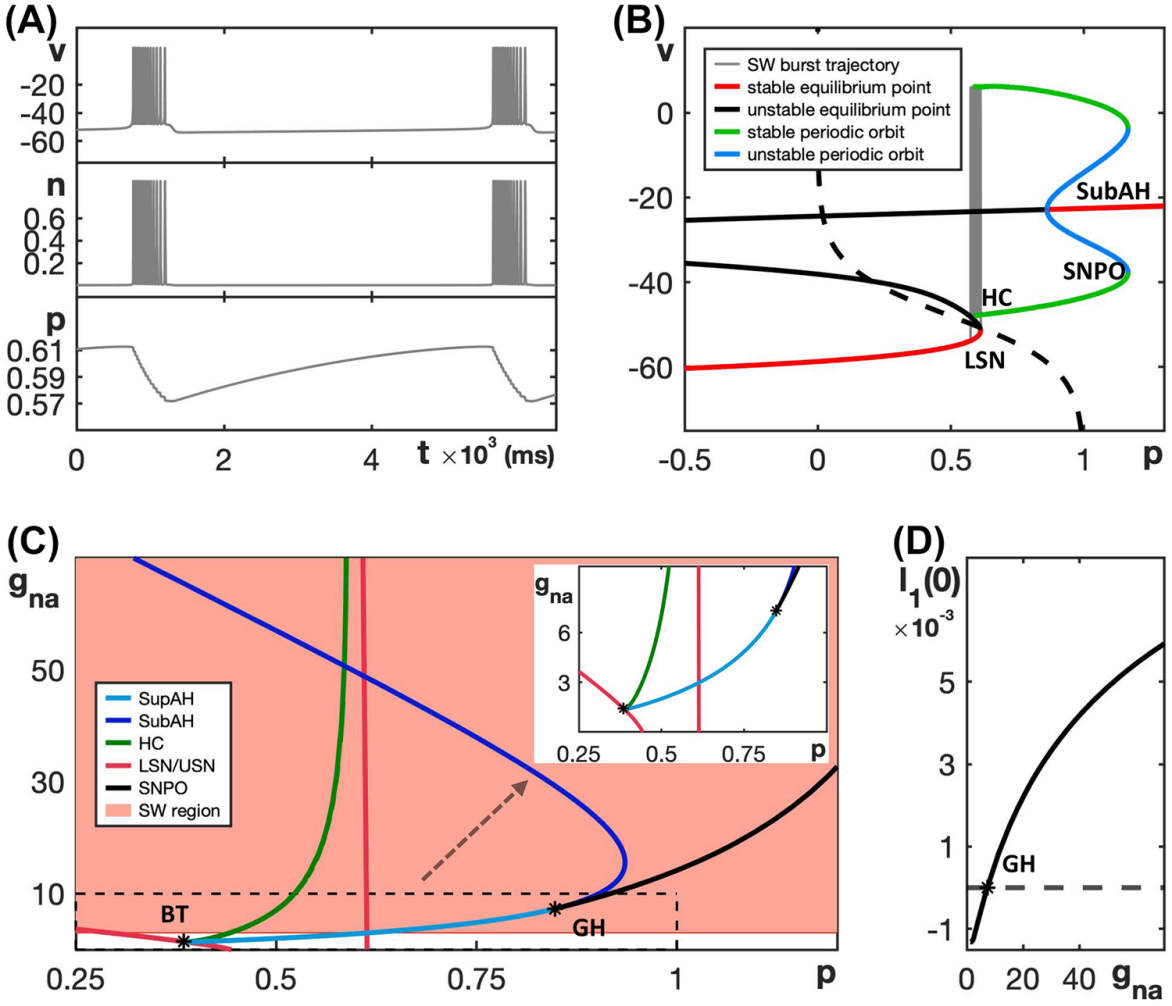
Dynamics and bifurcation structure for the Butera model (9) and (10). **A** SW bursting for the default parameters given in Table 4. **B** Bifurcation diagram of the model’s fast subsystem with respect to the slow variable *p*, together with the SW burst trajectory for the default parameters given in Table 4 overlaid in gray (evolution is counterclockwise). **C** Two-parameter bifurcation diagram of the fast subsystem in the $(p, g_{na})$-plane; colors are as in Fig. 3A and the SW bursting region is shaded red. The inset is an enlargement of the indicated region near BT and GH. **D** Lyapunov coefficent along the curve AH, comprised of SupAH and SubAH. The Andronov–Hopf bifurcation is supercritical only for $g_{na} < 7.3$, but a saddle-node of periodic orbits SNPO occurs at GH that generates a family of stable periodic orbits necessary for SW bursting

We choose default parameters as given in Table 4, such that the model exhibits SW bursting as shown in Fig. 8A.

**Table 4:** Butera Model (9) and (10): default parameter values

$c_m$	21 pF	$g_l$	2.8 nS
$g_{na}$	28 nS	$g_k$	11.2 nS
$g_{nap}$	2.8 nS	$g_{ton}$	0.3 nS
$g_{syn}$	0 nS	$e_l$	$-65$ mV
$e_{na}$	50 mV	$e_k$	$-85$ mV
$e_{syn}$	0 mV	$v_n$	$-29$ mV
$v_m$	$-34$ mV	$v_{mp}$	$-40$ mV
$v_p$	$-48$ mV	$s_m$	$-5$ mV
$s_n$	$-4$ mV	$s_p$	6 mV
$s_{mp}$	$-6$ mV	$\tau _n$	10 ms
$\tau _p$	10000 ms		

Non-dimensionalization (see the Appendix) shows that the timescale constants of *v*, *n* and *p* are $R_v \approx 1.33$, $R_n \approx 0.17$ and $R_p \in [0.0001,0.003]$, respectively. Decreasing $g_{na}$ decreases $R_v$, but $R_p$ remains much smaller than $R_v$ and $R_n$. Hence, we can again consider *v* and *n* as fast variables, with *p* the slow variable for this model.

Figure 8B shows the bifurcation diagram of the fast subsystem for the default parameter values. Observe that, even though the Andronov–Hopf bifurcation is subcritical, a family of stable periodic orbits (blue) originates from a saddle-node bifurcation of periodic orbits (SNPO). The SW burst trajectory (gray) is overlaid in Fig. 8B and evolves counterclockwise.

Figure 8C shows the two-parameter bifurcation diagram for the fast subsystem in the $(p, g_{na})$-plane. Notice that, in this figure, the order for the curves LSN and USN is reversed compared to the other models; compare also with Fig. 8B, where USN occurs at a negative value of *p* and is, hence, not visible in the view that is shown. For the Butera model (9) and (10), the SW bursting region (shaded red) persists as $g_{na}$ increases, because there always exists a family of stable periodic orbits of the fast subsystem in the region bounded by the curves HC and LSN. Indeed, even though the Andronov–Hopf bifurcation (blue) changes criticality at GH and is subcritical for $g_{na} > 7.3$ (Fig. 8D), the curve SNPO of saddle-node bifurcation of periodic orbits that emanates from GH persists and stays to the right of LSN (leftmost red curve). Hence, the fast subsystem always features a family of stable oscillations, which originate from SNPO and end (as *p* decreases) at HC (green). These stable oscillations support spiking in the active phase.

### Model modifications to include slow negative feedback

The biophysical mechanisms behind spiking for the canonical Hodgkin–Huxley model (Hodgkin & Huxley, 1952) include (i) a fast activating, more slowly inactivating inward sodium current that results in the upstroke of the spike and (ii) an outward, negative feedback potassium current, which activates on a timescale similar to that of the sodium inactivation and is responsible for the downstroke of the spike  (Tabak et al., 2011). Typical bursting models feature (i) and (ii) and also add in a third, slowest current or variable that helps to modulate the burst between its active and silent phases (Izhikevich, 2007). These components arise, in particular, in the neural and endocrine models presented in Section 2.1, which exhibit SW bursting in some region of parameter space. In the case of the generic endocrine model (3) and (4), for example, the calcium current $I_{Ca}$ is an inward current with fast activation and, hence, provides fast positive feedback to *v*, while the potassium current $I_K$ is an outward current with a slower activation gate *n* that provides the slow negative feedback; moreover, the slowest variable *c*, corresponding to the calcium concentration in the cell, modulates the burst.

Compared to the other models presented in Section 2.1, the Butera model (9) and (10) maintains SW bursting over a broad range of parameter values, as can be seen in Fig. 8C (red shaded region). The fast current in the Butera model is a sodium current, which is different from the fast calcium currents in the generic endocrine and minimal Chay–Keizer models, as well as the sodium current in the sodium-potassium minimal model, because $I_{Na}$ has a slow inactivation gate in this model. Past work has highlighted the roles of positive and negative feedback terms in tuning the features of neural spiking (Franci et al., 2013) and the potential importance of slow positive feedback in enhancing the robustness of bursting (Franci et al., 2018).

In this vein, we hypothesized that the robustness of SW bursting in the Butera model could relate to the fact that the additional negative feedback present in the model is slow relative to the fast activation. To test this idea, we modified each of the other three models to include a slow, voltage-dependent inactivation gate as part of the fast inward current, which allowed us to study how the inclusion of such a component alters each model’s dynamics.

The modified calcium current $I_{Ca}$ for the generic endocrine model (3) and (4) is given by

11 $$\begin{aligned} I_{Ca}(v) = g_{ca} \, m_{\infty }^2(v) \, h \, (v - e_{ca}), \end{aligned}$$

and the modified sodium current $I_{Na}$ for the sodium-potassium minimal model (5) and (6) takes the form

12 $$\begin{aligned} I_{Na}(v) = g_{na} \, m_{\infty }(v) \, h \, (v - e_{na}), \end{aligned}$$

where *h* in equations (11) and (12) is the voltage-dependent inactivation gating variable governed by the equation

13 $$\begin{aligned} h' = (h_{\infty }(v) - h)/\tau _h \end{aligned}$$

with

$$\begin{aligned} h_{\infty }(v) = (1 + e^{(v_h - v)/s_h})^{-1}. \end{aligned}$$

In its original form, the minimal Chay–Keizer model (7)–(8) has a fast inactivation term $h = h_{\infty }(v)$ associated with $I_{Ca}$. So to study this model, we changed the inactivation to a slow one by modifying the calcium current to take the form

14 $$\begin{aligned} I_{Ca}(v) = g_{ca} \, m_{\infty }^3(v) \, h \, (v - e_{ca}), \end{aligned}$$

where *h* again evolves according to equation (13).

Since *h* is a dimensionless variable that takes values between 0 and 1, the coupling of the *h*-dynamics via equations (11), (12) or (14) does not affect the timescale constants of the other variables in the models. The timescale constant for (13) is $R_h \approx Q_t/\tau _h = 1/\tau _h$, which can be derived similarly to the timescale of *n* (see the Appendix). For each model, we will explore how the dynamics and bifurcation structure change as $\tau _h$ is varied over a range of values. In each case, we choose this range to be roughly comparable with the model’s respective $\tau _n$-value, such that *h* and *n* evolve on similar timescales.

For the modified generic endocrine model (3), (4), (11), and (13), the half-inactivation value $v_h$ was selected to be $-30$, which is approximately in the middle of the range of *v*-values corresponding to the spiking phase of SW bursting in Fig. 2A. For simplicity, $s_h$ was kept constant at $-1$. In the Butera model (9) and (10), the inactivation of $I_{Na}$ is approximated as $1 - n$ where *n* is the activation variable of $I_K$. Following this idea, $v_h$ and $s_h$ of the modified sodium-potassium minimal model (5), (6), (12), and (13) were chosen as $-25$ and $-5$, respectively, to match the values associated with corresponding terms for $I_K$ in that model. For the modified minimal Chay–Keizer model (7), (8), (13), and (14), we retained the default definitions and parameter values for $h_{\infty }(v)$ given in Table 3.

## Results

In this section we investigate the robustness of SW bursting to variation of the fast inward current conductance for each of the three modified models. We first analyze how this robustness depends on the the timescale constant $\tau _h$. For the values that we include, all of the modified models can be considered as fast-slow systems with fast variables *v*, *n*, and *h* and a single slow variable. Hence, we can apply a similar fast-slow analysis to these modified models to that employed in Section 2. We also consider the effect of varying the conductance $g_k$ associated with the potassium current that is present in all four models and complete our results with a two-parameter analysis with respect to $g_k$ and the inverse $\tau _h^{-1}$ of the timescale constant.

### Bifurcation diagrams of modified models

**Fig. 9:**
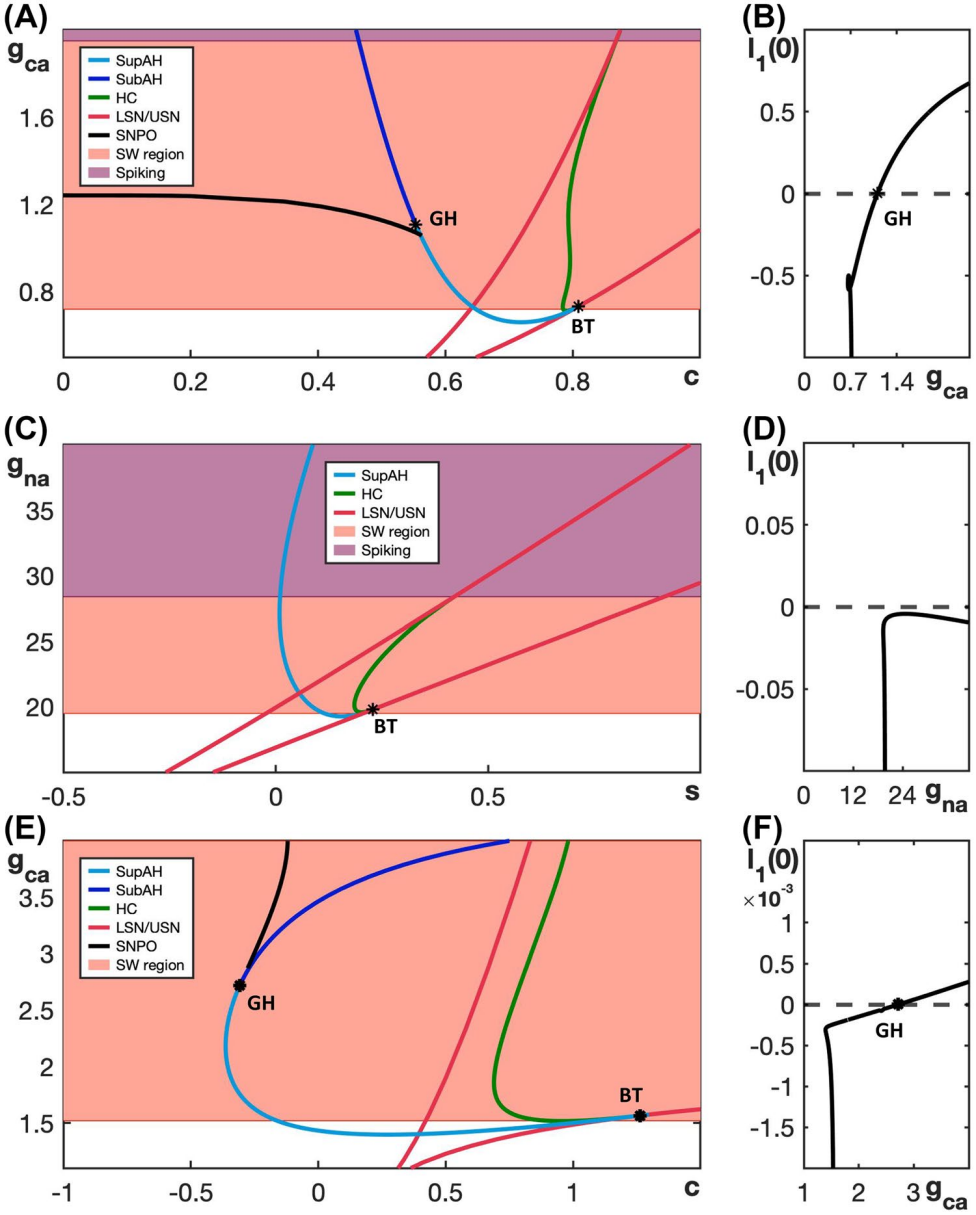
Two-parameter bifurcation diagram of the fast subsystem for each of the three modified models with respect to the corresponding slow variable and the conductance of its fast inward current. **A** Modified generic endocrine model (3), (4), (11), and (13). **B** Lyapunov coefficent associated with the curve AH (composed of SupAH and SubAH) in (**A**), plotted versus $g_{ca}$ with $\tau _h=0.033$ . **C** Modified sodium-potassium minimal model (5), (6), (12), and (13) with $\tau _h=0.125$. **D** Lyapunov coefficent associated with the curve SupAH in (**C**), plotted versus $g_{na}$. **E** Modified minimal Chay–Keizer model (7), (8), (13), and (14) with $\tau _h=1.111$. **F** Lyapunov coefficent associated with the curve AH in **(E)**, plotted versus $g_{ca}$. All of the modified models show broader parameter ranges over which they exhibit SW bursting (shaded red) compared to those in Figs. 3, 5, and 7 for the unmodified models. Moreover, unlike all of the originals, none of the modified models yield transitions to PP bursting or depolarization block

Figure 9 shows two-parameter bifurcation diagrams of the respective fast subsystem for each of the three modified models for a fixed $\tau _h$ value; here, we plot the slow variable (*c* or *s*) on the horizontal and the conductance of fast inward current ($g_{ca}$ or $g_{na}$) on the vertical axis. These bifurcation diagrams should be compared to Figs. 3, 5, and 7, respectively. Notice that in all of these diagrams, the curve AH of Andonov–Hopf bifurcations is pushed out far to the left of the leftmost saddle-node curve LSN as $g_{ca}$ or $g_{na}$ increases. This arrangement of bifurcations ensures the existence of a family of stable periodic orbits in the fast subsystem over a larger range of $g_{ca}$ or $g_{na}$, which prevents a transition to PP bursting. Instead, the pattern exhibited in each case is either SW bursting or slow spiking, depending on the position of the curve HC of homoclinic bifurcations. Specifically, if the curve HC does not reach the curve LSN and the Andronov–Hopf bifurcation is supercritical (or subcritical with a curve SNPO of saddle-node bifurcations of periodic orbits located even farther away from LSN), then SW bursting results. On the other hand, if the homoclinic curve reaches LSN, which induces a so-called SNIC regime (saddle-node bifurcation on invariant cycle) (Ermentrout, 1996), then the model exhibits slow spiking. In all of the two-parameter diagrams shown in Figure 9, the SW and slow spiking regions are shaded red and purple, respectively.

The bifurcation diagram for the modified generic endocrine model (3), (4), (11), and (13) in the $(c, g_{ca})$-plane with $\tau _h=0.033$ (Fig. 9A) shows a clear expansion in the range of $g_{ca}$-values for which the model exhibits SW bursting relative to the original model (cf. Fig. 3). Indeed, when the Andronov–Hopf bifurcation switches from supercritical to subcritical for $g_{ca}$ just above 1 (Fig. 9B), a saddle-node bifurcation of periodic orbits (SNPO) occurs to the left of the curve AH and therefore stable oscillations persist, extending in the direction of increasing *c* until the curve HC is reached. Consequently, a transition to PP bursting is now prevented. Instead, when SW bursting is lost for $g_{ca}$ just below 2, due to the transition from HC to SNIC, the modified model generates slow spiking.

In the case of the modified sodium-potassium minimal model (5), (6), (12), and (13), the two-parameter bifurcation diagram in the $(s, g_{na})$-plane with $\tau _h=0.125$ (Fig. 9C) features a slightly expanded SW bursting range. Recall that SW bursting for the original sodium-potassium minimal model (Fig. 5B) is not especially robust to parameter changes. Even though SW bursting never transitions to PP bursting for the original form of this model, when $g_{na}$ is increased above the SW range, the original model exhibits some intermediate patterns and then transitions to a stable steady state of the full system at elevated voltage. In the modified model, on the other hand, when SW bursting is lost, the system switches to slow spiking through the transition to a SNIC (Fig. 9C). There is also no longer a change in criticality of the Andronov–Hopf bifurcation in this modified model, at least not over the range of $g_{na}$-values considered (Fig. 9D).

The modified minimal Chay–Keizer model (7), (8), (13), and (14) with $\tau _h=1.111$ shows an expansion in its SW region in the $(c, g_{ca})$-plane relative to the original version of the model (compare Fig. 7 with Fig. 9E) and, similarly to the other modified models, it no longer supports PP bursting. Instead, the SW regime features a curve SubAH of subcritical Andronov-Hopf bifurcation (Fig. 9F), with an associated family of stable periodic orbits originating at the curve SNPO. Although these bifurcation curves lie at non-physiological, negative *c*-values, the stable periodic orbits extend to positive *c* and terminate at the curve HC in the SW bursting regime. The modified model transitions directly from SW bursting to spiking as $g_{ca}$ is increased, organized by the switch to the SNIC mechanism.

**Fig. 10:**
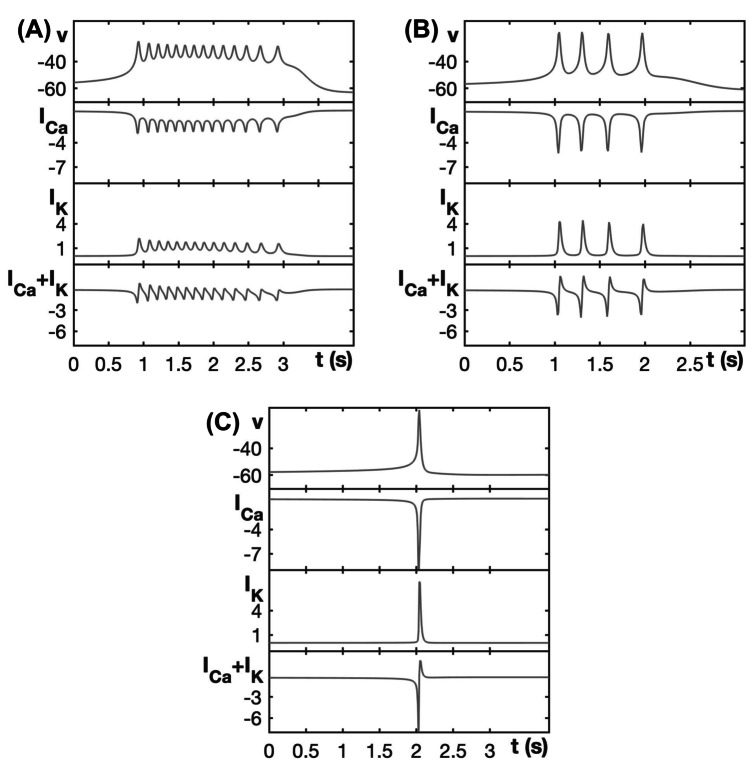
Burst patterns exhibited by the modified generic endocrine model (3), (4), (11), and (13) for different values of $g_{ca}$, along with associated currents. **A** SW bursting at $g_{ca} = 0.81$. **B** SW bursting at $g_{ca} = 1.2$. **C** Spiking for $g_{ca} = 2.0$. Note that, as $g_{ca}$ increases, the amplitude of the initial spike increases

Figure 10 compares the burst patterns of the modified generic endocrine model (3), (4), (11), and (13) for progressively increasing values of $g_{ca}$. The first two panels are very similar to those of Fig. 4 for the unmodified model. With the modification, *h* can decay on each spike, but that has little qualitative impact on burst features for these $g_{ca}$-values. Once $g_{ca}$ becomes large enough that PP bursting would have occurred in the original model, however, a more significant difference emerges (Fig. 10C). In this case, the large $g_{ca}$ and corresponding $I_{Ca}$ yield a strong voltage elevation and $I_K$ activation as previously. However, when the strong $I_K$ activation is combined with *h* inactivation that weakens $I_{Ca}$, the outward current overwhelms the inward current, as can be seen from the positive value of $I_K + I_{Ca}$ on the tail end of the spike. Hence, *v* is now pushed down to a hyperpolarized state, and bursting is replaced by the generation of an isolated spike.

### Effects of varying $\tau _h$

As we and many others have discussed (Ermentrout & Terman, 2010; Izhikevich, 2007), bursting in neuronal and endocrine models relies on a balance of voltage-dependent positive and negative feedback contributions to the voltage equation, acting on appropriate timescales. More specifically, consider SW bursting in a model for which the fast inward current does not inactivate. If the conductance of this inward current is increased sufficiently then the strengthened positive feedback disrupts the balance of currents in the system. As a consequence, the slower negative feedback current cannot overcome the fast positive current to induce the downstroke needed for a spike, so the model ceases to exhibit spiking during its active phase and, instead, transitions to a state of depolarization block or a PP burst. Therefore, we hypothesize that the enhancement of SW bursting, and the prevention of PP bursting and depolarization block, can be achieved by modifications to a model in such a way that the balance of currents is maintained as certain parameters vary. We achieved this by adding a slow inactivation gate to a positive current, such that this inward current gradually weakens, even when its maximal conductance $g_{ca}$ or $g_{na}$ is high. In this section, we report on achieving an optimal balance by choosing the most suitable value for $\tau _h$, the time constant for the slow inactivation gate.

**Fig. 11:**
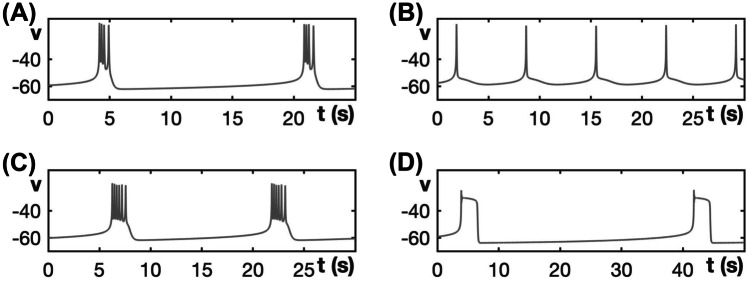
Activity patterns exhibited by the modified generic endocrine model (3), (4), (11), and (13) at $g_{ca} = 1.1$ with $\tau _h$ varying. **A** SW bursting for the original model, which is equivalent to the modified model with $\tau _h = \infty$ and $h \equiv 1$. **B** Slow spiking for $\tau _h = 0.2$. **C** SW bursting for the default value $\tau _h = 0.03$. **D** PP bursting for $\tau _h = 0.01$

Figure 11 shows the burst patterns of the original generic endocrine model (3) and (4) as well as of its modification with (11) and (13) for $g_{ca} = 1.1$ and different values of $\tau _h$. When $g_{ca} = 1.1$, the original model, without inactivation of the $Ca^{2+}$-channel, exhibits SW bursting (Fig. 11A); this corresponds to setting $\tau _h = \infty$, with $h \equiv 1$ constant, in the modified generic endocrine model. Note that the value $g_{ca} = 1.1$ is at the top end of the $g_{ca}$-range at which the original model can potentially produce SW bursting (cf. Fig. 3). We now impose dynamics on the inactivation gate to the calcium channel and show how the balance of voltage-dependent positive and negative feedback is controlled by the timescale constant $\tau _h$ associated with this inactivation gate.

When $\tau _h=0.2$ (Fig. 11B), the dynamics of *h* is not fast enough during the first spike to cause any spike attenuation. Hence, *v* reaches a level at which the outward current $I_K$ turns on to full strength. As the spike terminates, the combination of the small decrease in *h* and corresponding decrease in $I_{Ca}$ together with the strong $I_K$ result in a net outward current flow that pulls the voltage back down out of the active phase into a full after-hyperpolarization. Thus, when the inactivation is slow, SW bursting is replaced by slow spiking.

Decreasing $\tau _h$ to the default value $\tau _h = 0.03$ for the modified model (as used in Fig. 9A) corresponds to a faster, although still slow, rate of change of *h*. In this case, *h* reduces fast enough that the amplitude of the first spike is lowered, as seen in Fig. 11C; indeed, notice that the spikes max out at a lower voltage than in Fig. 11A, B. The reduced maximal voltage leads to a weaker $I_K$ activation, which cannot induce a full hyperpolarization or return to the silent phase. Hence, additional spikes occur, even though *h* is gradually decreasing, resulting in a spiking active phase and restoration of a SW bursting pattern.

Decreasing $\tau _h$ further, however, accelerates the $I_{Ca}$ inactivation rate, which means that the amplitude of the first voltage peak is lowered even more and, consequently, $I_K$ activation is significantly weakened. Eventually, the outward $I_K$ current is not strong enough to pull down the voltage and form a spike. This effect corresponds to convergence to the depolarized (upper) branch of the critical manifold. Hence, voltage jumps up to the branch of $\mathcal {C}$ with stable equilibria of the fast subsystem, which leads to transient depolarization block and the emergence of PP bursting patterns (e.g., Fig. 11D for $\tau _h = 0.01$), or else sustained depolarization block.

**Fig. 12:**
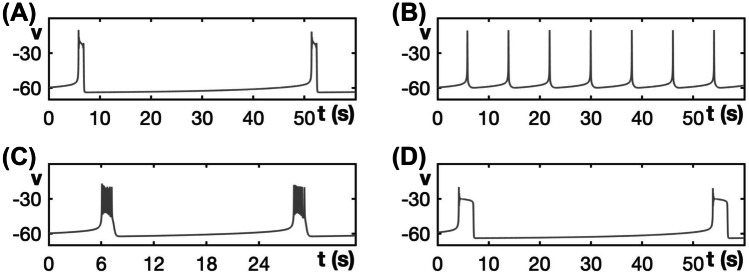
Activity patterns exhibited by the modified generic endocrine model (3), (4), (11), and (13) at $g_{ca} = 1.5$ with $\tau _h$ varying. **A** PP bursting for the original model, which is equivalent to $\tau _h = \infty$ and $h \equiv 1$. **B** Slow spiking for $\tau _h = 0.2$. **C** SW bursting for $\tau _h = 0.02$. **D** PP bursting for $\tau _h=0.015$

If we now increase $g_{ca}$ to $g_{ca} = 1.5$ then the original generic endocrine model (3) and (4) exhibits PP bursting (cf. Fig. 3); the burst pattern is shown in Fig. 12A, with the other panels illustrating burst patterns for the modified generic endocrine model (3), (4), (11), and (13) with $g_{ca} = 1.5$ and different values of $\tau _h$. We select $\tau _h = 0.2$ (Fig. 12B), $\tau _h = 0.02$ (Fig. 12C), and $\tau _h = 0.015$ (Fig. 12D), which produce a sequence of patterns that suggest a similar transition from spiking via SW bursting to PP bursting (cf. Fig. 11), even though the original model exhibits only PP bursting at this higher $g_{ca}$-value. The explanation is entirely analogous to that detailed for Fig. 11; for example, when $\tau _h = 0.2$, the inactivation gate is very slow and *h* does not change enough during the first spike to cause any reduction in peak spike amplitude. With the slow inactivation of $I_{Ca}$, however, the resulting increase in $I_K$ is strong enough to pull the voltage back to full hyperpolarization after the first spike.

**Fig. 13:**
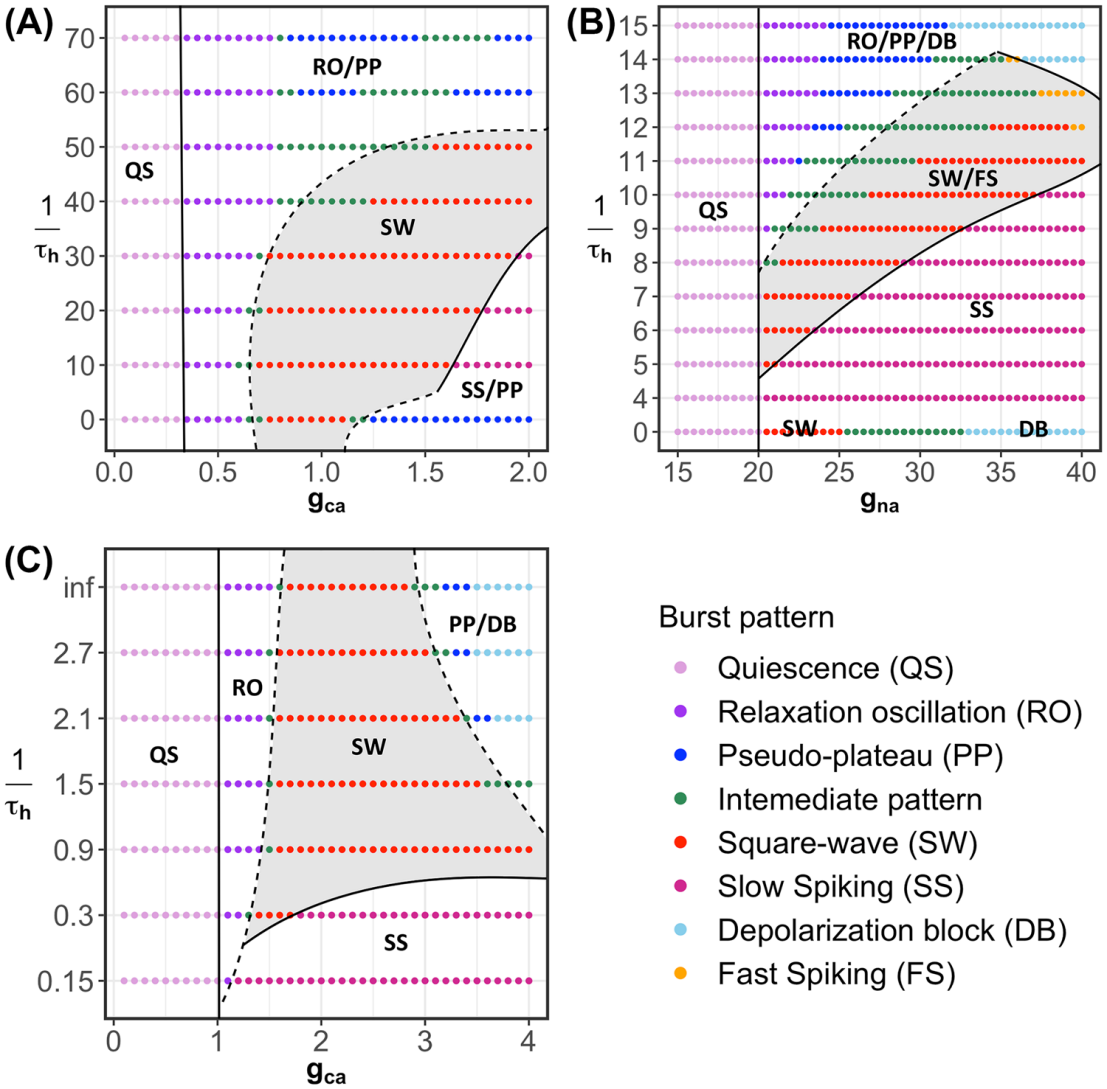
Two-parameter bifurcation diagrams of the modified models with respect to $g_{ca}$ or $g_{na}$ and $1/\tau _h$. **A** Modified generic endocrine model (3), (4), (11), and (13). **B** Modified sodium-potassium minimal model (5), (6), (12), and (13). **C** Modified minimal Chay–Keizer model (7), (8), (13), and (14). Notice that in all the three modified models, SW is most robust over an intermediate range of $1/\tau _h$-values (and, hence, of $\tau _h$-values)

We observe the same effect when varying $\tau _h$ for different choices of $g_{na}$ in the modified sodium-potassium minimal model and for different choices of $g_{ca}$ in the modified Chay-Keizer model. In other words, for all three modified models, there exists an intermediate range of $\tau _h$-values for which the SW burst regime is significantly extended into higher values for $g_{ca}$ or $g_{na}$ and PP bursting is prevented. Figure 13 illustrates this enlarged robustness with two-parameter bifurcation diagrams of all three modified models that show the regimes for different activity patterns with respect to the conductance of the fast inward current, $g_{ca}$ or $g_{na}$, and $1/\tau _h$. We use the inverse $1/\tau _h$ rather than $\tau _h$ itself so that the activity patterns of the original generic endocrine model (3) and (4) and the original sodium-potassium minimal model (5) and (6) appear on the line $1/\tau _h = 0$. For the minimal Chay–Keizer model (7) and (8), the inclusion of $h_{\infty }(v)$ in $I_{Ca}$ corresponds to an instantaneous negative feedback component of this current. Therefore, this model is represented as $1/\tau _h=\infty$ (“inf”) in Fig. 13C.

For each fixed value of $\tau _h$, the activity patterns exhibited by the modified models were analyzed by considering two-parameter bifurcation diagrams with respect to the fast inward current conductance parameter and the slow variable, as in earlier figures (e.g., Fig. 3). In each panel, the gray shaded region corresponds to SW bursting or fast spiking patterns, both of which would yield synaptic transmission. Fast spiking is exhibited by the modified sodium-potassium minimal model (5), (6), (12), and (13) for $\tau _h$ values above 11 and sufficiently large $g_{na}$. In this case, the full model has a stable periodic orbit with $s\approx 1$; for example, see Supplemental Fig. 2. Observe that, for all three modified models, the largest interval of conductances that spans this region occurs at the cross-section for an intermediate value of $1/\tau _h$ (and, thus, of $\tau _h$). Indeed, for Fig. 9, we selected $\tau _h$ values near the optimum for each model. When $\tau _h$ is sufficiently small, all three modified models exhibit relaxation oscillations that transition to PP bursting as the fast inward current conductance is increased. From there, as $\tau _h$ is made larger, an interval of conductances that support SW bursting emerges and grows (Fig. 13A–C) and PP bursting, over a large range of $\tau _h$, is prevented.

We remark that the analysis of the corresponding bursting patterns for most of the range of $\tau _h$-values considered can be done by assuming that the model has three fast and one slow variables. However, at sufficiently large values of $\tau _h$, the timescale of *h* becomes comparable to that of the slow variable, which means that the models should be analyzed as systems with two fast and two slow variables. Our numerical explorations for each of the three modified models suggest that on the intermediate range of $\tau _h$ that extends the SW regime, $\tau _h$ does not yet become comparable to the timescale of the slow variable. We leave a more detailed multi-timescale analysis of the regime of large $\tau _h$ for future work.

### Varying $\varvec{g_k}$  

Varying the parameter $g_k$ changes the timescale of *v* but leaves the timescales of the other variables unchanged (see the Appendix). Hence, the modified models can be analyzed for varying $g_k$ by considering a fast-slow decomposition with three fast and one slow variables, as long as *v* remains fast, and also $\tau _h$ for each modified model is chosen such that the *h*-kinetics evolves at a significantly faster timescale than that of the slowest variable in the corresponding original model.

**Fig. 14:**
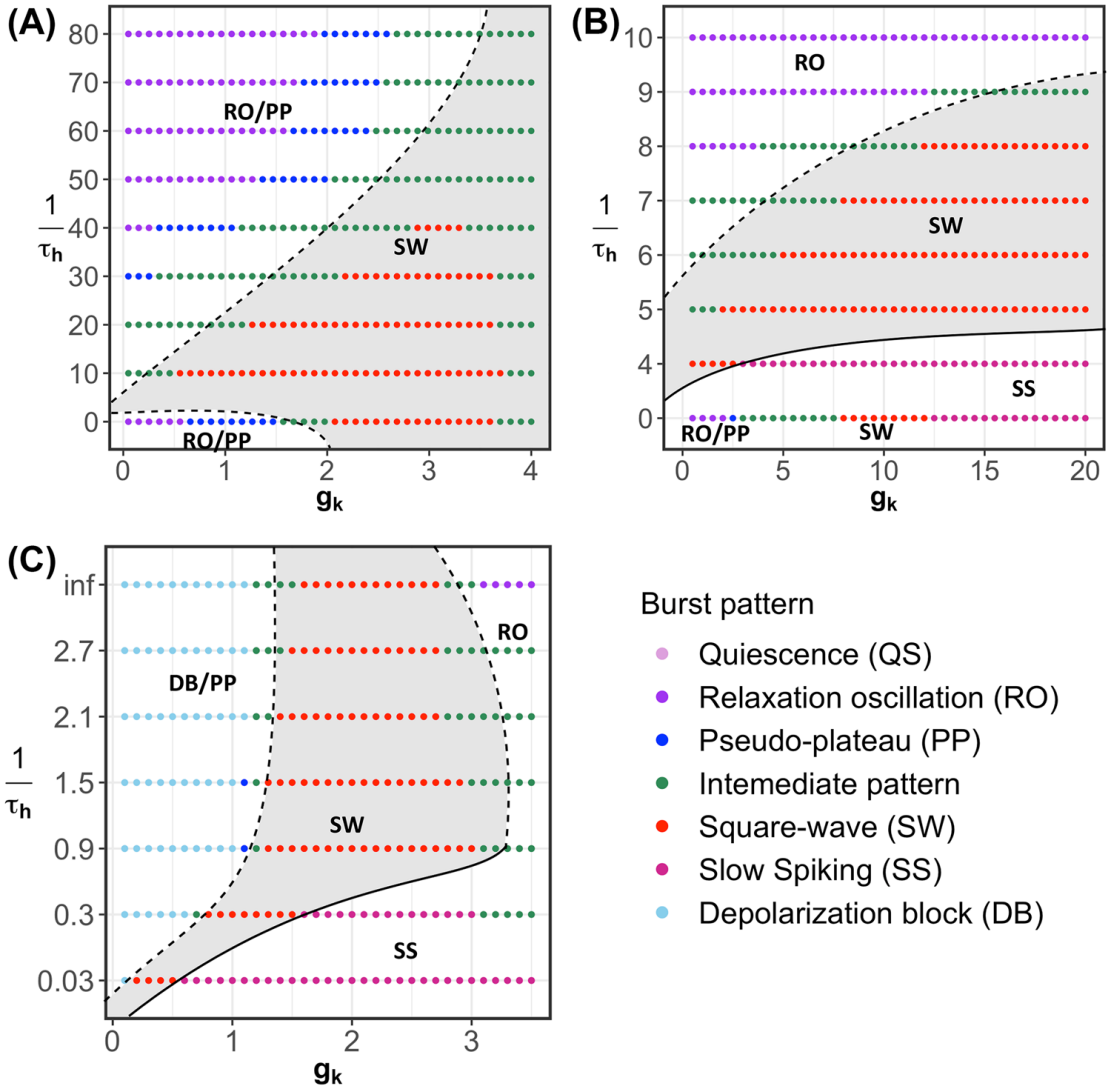
Two-parameter bifurcation diagrams of the modified models with respect to $g_k$ and $1/\tau _h$. **A** Modified generic endocrine model (3), (4), (11), and (13). **B** Modified sodium-potassium minimal model (5), (6), (12), and (13). **C** Modified minimal Chay–Keizer model (7), (8), (13), and (14)

For a general SW bursting model, a reduction of $g_k$ leads to a transition from SW bursting to a PP pattern; qualitatively, it has the same impact as increasing $g_{na}$ or $g_{ca}$ (Teka et al., 2011b). Therefore, we expect that the robustness of SW bursting with respect to changes in $g_k$ is maximal for the modified models if $\tau _h$ is chosen from an intermediate range. This is confirmed in Fig. 14, where we show two-parameter bifurcation diagrams in the $(g_k, 1/\tau _h)$-plane for each of the modified models.

Figure 14A corresponds to the modified generic endocrine model (3), (4), (11), and (13). Notice that in the original model, without inactivation (i.e., $1/\tau _h = 0$), the burst pattern transitions to PP (blue dots) as $g_k$ decreases below $g_k \approx 1.7$. Over a range of $1/\tau _h$-values that are neither too large nor too small, this transition is completely prevented. The modified sodium-potassium minimal model (5), (6), (12), and (13) in Fig. 14B yields a qualitatively similar result. Recall that the original minimal Chay–Keizer model (7) and (8), with its instantaneous $I_{Ca}$ inactivation gate, corresponds to $1/\tau _h = \infty$ (“inf”) in Fig. 14C; the burst pattern transitions to depolarization block (light blue) as $g_k$ decreases, via only a very small interval of PP activity. The modified minimal Chay–Keizer model with additional Eqs. (13) and (14) maintains this property for large $1/\tau _h$-values, but the transition via PP to depolarization block is prevented over a much larger range of $g_k$ for an intermediate interval of $1/\tau _h$-values.

### Effect of slow negative feedback on the location of AH

The additional inactivation gate and associated *h*-dynamics affect the location of the critical manifold for the fast subsystem (2), but this location change does not fully explain the increased robustness seen at intermediate values of the timescale constant $\tau _h$. For example, consider the modified generic endocrine model (3), (4), (11), and (13) for the default parameters as given in Table 1, with various choices of $\tau _h$. Provided that $\tau _h$ remains small enough, e.g., $\frac{1}{\tau _h} > 10$, the modified model has three fast variables (*v*, *n* and *h*) and one slow variable (*c*). Then the critical manifold is defined implicitly by the equation:

15 $$I_{Ca}(v, h_{\infty }(v)) + I_K(v, n_{\infty }(v)) + I_{K(Ca)}(v,c) = 0,$$

with $n = n_{\infty }(v)$ and $h = h_{\infty }(v)$. Hence, the critical manifold does not depend on $\tau _h$ at all. Similarly, the saddle-node bifurcations LSN and USN, which are determined by the local minima and maxima of (15), respectively, when viewed as a curve in the (*v*, *c*)-plane, do not depend on $\tau _h$. However, the Jacobian matrix of the full four-dimensional system, evaluated along the critical manifold, does depend on $\tau _h$; this means, in particular, that the location of the Andronov–Hopf bifurcation (AH) is potentially affected by variations in $\tau _h$.

**Fig. 15:**
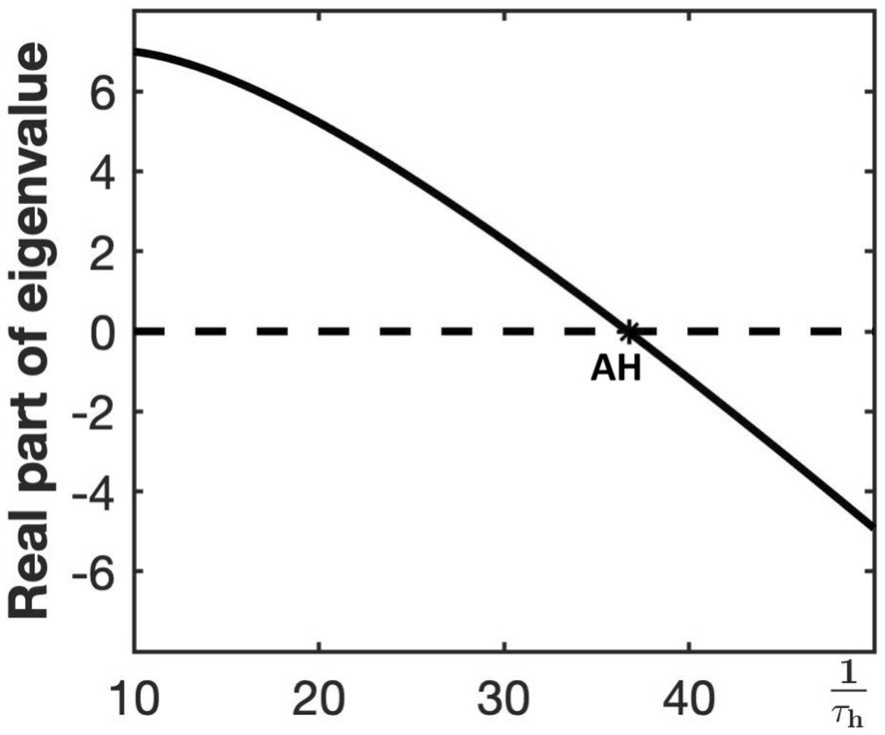
Dependence on $\tau _h$ of two complex-conjugate eigenvalues associated with an equilibrium of the fast subsystem with $c > c_\textsf{LSN}$ on the upper branch of the critical manifold  $\mathcal {C}$ for the modified generic endocrine model (3), (4), (11), and (13) with $g_{ca} = 0.81$. Shown are their real parts versus $1/\tau _h$. The stability changes at an Andronov–Hopf bifurcation (AH) for an intermediate value of $1/\tau _h$. Hence, below this value, the system will not exhibit depolarization block or PP bursting

For example, consider an equilibrium of the fast subsystem that lies on the upper, high-voltage branch of the critical manifold $\mathcal {C}$ on the part that coexists with its middle branch and (part of) its lower branch; hence, its *c*-coordinate satisfies $c_\textsf{LSN} \le c \le c_\textsf{USN}$. Figure 15 shows how the real parts of a pair of complex-conjugate eigenvalues for this equilibrium point change with $1/\tau _h$ for fixed $g_{ca} = 0.81$. As can be seen from the figure, for relatively small $\tau _h$ values (i.e., for large $1/\tau _h$), the equilibrium is stable. That is, the Andronov–Hopf bifurcation, denoted AH here, that stabilizes points on the upper branch of  $\mathcal {C}$ occurs at a *c*-value above the *c*-coordinate of this equilibrium point. On the other hand, as $\tau _h$ becomes larger, corresponding to a slower negative feedback, the equilibrium becomes unstable. In this case, the Andronov–Hopf bifurcation point AH must occur at a lower *c*-value than that of this equilibrium point. For a model to exhibit PP bursting or depolarization block, the point AH must lie at $c > c_\textsf{LSN}$. Increasing $\tau _h$ pushes this bifurcation to *c-*values below $c_\textsf{LSN}$; that is, the calcium inactivation must be sufficiently slow to move the Andronov–Hopf bifurcation AH to a location where PP bursting and depolarization block are prevented for this value of $g_{ca}$.

## Discussion

In this work, we compared bursting patterns across four well-established, relatively low-dimensional mathematical neuron models of Hodgkin–Huxley type, namely, a generic endocrine model (3) and (4) (Tsaneva-Atanasova et al., 2010), a sodium-potassium minimal model (5) and (6) (Izhikevich, 2007), a minimal Chay–Keizer model (7) and (8) (Rinzel, 1986; Rinzel & Lee, 1986), and the Butera model (9) and (10) (Butera et al., 1999). Observing the distinctive robustness of SW bursting in the Butera model, which features a slow inactivation component in the fast inward current that drives spiking, we modified the three other models, which exhibit less robust SW bursting in their original forms. Specifically, we included slow inactivation dynamics in their fast inward currents, and examined the effects on the robustness of their SW bursting dynamics. Previous literature has studied the transition between SW and PP bursting patterns with changes in fast inward current conductances (Osinga et al., 2012; Tabak et al., 2007; Teka et al., 2011a, b; Tsaneva-Atanasova et al., 2010). To our knowledge, however, this is the first time that the effect of slow negative feedback has been studied in relation to the robustness of SW bursting. The point of this analysis is not to propose an adjustment to these bursting models; rather, we use the comparison of the original and modified models as a tool to explore the role of the slow inactivation of the inward current. Our results provide insight into why some neurons in biological systems might have slowly inactivating inward currents, despite their seeming redundancy because of the presence of outward currents that activate on similar timescales.

We employed standard dynamical systems methods of fast-slow decomposition and bifurcation analysis for this investigation. Our analysis shows that the addition of slow inactivation dynamics expands the ranges of parameter values over which the modified models exhibit SW bursting, while eliminating or curtailing PP bursting, depolarization block, and relaxation oscillations. This finding led us to the novel hypothesis that inward currents featuring slow inactivation should be prevalent for neurons that rely on bursts with spikes for synaptic transmission and the activation of associated calcium currents (e.g., Phillips et al. 2019).

The bifurcation techniques and fast-slow analysis used in this work depend heavily on the timescale separation of the variables in these models. We showed that the modified models exhibit optimally robust SW bursting if the timescale constant associated with the inward current inactivation lies in a range that is similar to that of the activation variable for a primary outward current (e.g., $I_K$). When the slow inactivation is too fast in these relative terms, we observed that the inward current can become too weak to recruit the outward current and induce the corresponding hyperpolarization needed to sustain repeated spiking, in which case patterns such as PP bursting are more likely (e.g., Fig. 13A, large $1/\tau _h$). This finding is analogous to the result that a fast-activating negative feedback provided by a BK potassium current promotes PP bursting in pituitary cells (Vo et al., 2014). When the slow inactivation is too slow, the inward current can become too strong, so that even with full outward current activation, the cell does not repolarize. Thus, there is a “Goldilocks zone” for tuning the timescale of the inward current inactivation where it is most effective at sustaining spiking and associated synaptic transmission.

We linked these ideas with specific mathematical properties of the models by studying how this inactivation rate affects the stability of equilibria in the fast subsystem at elevated voltage and the location in parameter space of the Andronov–Hopf bifurcation points at which these equilibria change stability. Here we made use of the fact that there is generally a single slow variable for the considered values or ranges of the relevant system parameters. It remains an interesting subject of future mathematical work to calculate bounds on the optimal range of inactivation timescales for maximal robustness of SW. This will likely require the consideration of parameter ranges where one finds two slow variables, in addition to ranges where there is just one. Another direction for future analysis would be to consider effects on robustness due to variation of other model parameters that are affected by neuromodulation or are relevant to pathologies involving alterations to neural bursting; for example, see (Goldman et al., 2001; Kubota & Rubin, 2011; Loucif et al., 2008; Städele & Stein, 2022).

The values for the half-inactivation parameters $v_h$ and $s_h$ in (13) for the models that we studied were chosen to match analogous values used in other models with inactivation gates for the inward current (Butera et al., 1999; Rinzel, 1986; Rinzel & Lee, 1986). Changing these values yields a quantitatively different optimal timescale range over which SW bursting is most robust, but our numerical explorations suggest that this does not change the phenomenon that we revealed (e.g., see Supplemental Fig. 4). We considered only four models that were known to exhibit SW bursting, two with a fast inward sodium current and two with a fast inward calcium current. Despite our focus on a small selection of models, the mechanistic aspects of the results that we have explained strongly suggest that our results will naturally generalize beyond these specific examples.

We note that the same fast subsystem bifurcation structure that supports SW bursting can also yield sustained, fast, tonic spiking, depending on the position of the slow nullcline (e.g., Supplemental Figs. 2 and 3). However, we found that the occurrence of this type of spiking is quite rare in the models that we studied, although it does show up in one case (Fig. 13 B, orange dots). In other models that include a slow negative feedback on the fast inward current, SW bursting could be lost to this fast spiking more commonly under parameter variation. In a CPG (central pattern generator) circuit, however, this activity could serve a similar function as SW bursting. To see this, suppose that two or more intrinsically spiking neurons are coupled by synaptic inhibition and one is actively spiking, leading to the inhibition of the others. If one of these other neurons becomes active, such as through recovery from adaptation, and starts spiking, then it could inhibit and shut off the formerly spiking neuron. When this process occurs repeatedly, it results in bursting spike patterns (cf. Rubin & Smith 2019). Interestingly, CPG circuits with reciprocal inhibition can exhibit phase transitions based on a release mechanism, controlled by neurons in the active phase, or an escape mechanism, controlled by neurons in the silent phase. In the former case, the synaptic threshold is likely to be elevated, such that spiking is important for circuit oscillation properties, whereas in the latter case, the synaptic threshold is likely to be lower, such that the presence of spikes within each phase of depolarized membrane potential becomes less important (Sharp et al., 1996); hence, our work suggests that the presence of inward currents with slow inactivation might be an indicator that a circuit operates in release mode.

Ideally, in future work, a more general theory can be developed that will cast our results in terms of assumptions on a general Hodgkin–Huxley type model. For the time being, we can at least observe that the results of this study are consistent with past work on neuronal bursting (Izhikevich, 2007) in that we also find that, for a neuron with slow inward current inactivation, it is not important whether sodium or calcium ions are carried in this current. Interestingly, however, a key prediction emerges: fast currents with slow inactivation, which are usually sodium currents, will represent the dominant, fast inward current in rhythmic neurons for which spiking is important; non-inactivating sodium currents and calcium currents or the presence of fast negative feedback (Vo et al., 2014), on the other hand, will tend to be associated with neurons for which spiking is less important than simple depolarization. Correspondingly, in neurons for which function is unknown, the characterization of the dominant, fast inward current gives us a prediction about the importance of spiking for these cells.

### Supplementary Information

Below is the link to the electronic supplementary material.

Supplementary file 1: **A** Depolarization block exhibited by the sodium-potassium  minimal model (5)-(6) resulting from a full-system stable steady state $(v, n, s) \approx (-11.5,0.93,1.0)$ at elevated voltage for $g_{na} = 35$. **B** Bifurcation diagram of the model's fast system associated with **(A)**. The stable steady state lies where the fast subsystem equilibrium curve intersects the *s*-nullcline (dashed black) (pdf 121 KB)

Supplementary file 2: **A** Fast spiking exhibited by the modified sodium-potassium  minimal model (5)-(6), (12)-(13) resulting from a full-system stable periodic orbit with $s \approx 1.0$ for $g_{na} = 39$ and $1/\tau_h=13$. **B** Bifurcation diagram of the model's fast system associated with **(A)** (pdf 258 KB)

Supplementary file 3: **A** Fast spiking exhibited by the generic endocrine model (3)-(4) resulting from a full-system stable periodic orbit with $c \approx 0.7$ for $g_{ca} = 0.81$ and $\alpha=2$. **B** Bifurcation diagram of the model's fast system associated with **(A)** (pdf 290 KB)

Supplementary file 4: Two-parameter bifurcation diagrams of the modified generic endocrine model (3)-(4), (11), and (13), with respect to **A** $g_{ca}$ and $1/\tau_h$ for $v_h=-30$ and $s_h=-1$; **B** $g_{ca}$ and $1/\tau_h$ for $v_h=-35$ and $s_h=-1$; **C** $v_h$ and $g_{ca}$ for $1/\tau_h=30$ and $s_h=-1$; **D** $s_h$ and $g_{ca}$ for $v_h=-30$ and $1/\tau_h=30$ (pdf 521 KB)

## Data Availability

No experimental data was used in this work. The computational techniques for this contribution are described in the article. Further inquiries can be directed to the authors.

## Appendix

Non-dimensionalization of a model is a form of scaling that expresses the rate of change of each model variable as the product of a dimensionless speed and a function of constrained magnitude. The dimensionless speeds are appropriate to be compared across all variables to evaluate their relative rates of change. In this process, each original variable is represented as a fraction of a nominal value, often taken to be the maximum of the range over which that variable is observed to evolve in the dynamics of interest. The equations are then expressed in terms of these non-dimensional fractions and the magnitudes of the corresponding unitless speeds represent their timescale constants; note that gating variables are already non-dimensional fractions with maximal values of one, so no scaling is needed for their equations. We derive the scaled equations in detail for the generic endocrine model (3) and (4); a similar derivation leads to the scaled equations for the other three models, which are merely stated for reference.

### Generic endocrine model

Note that the variable *n* for the generic endocrine model (3) and (4) is a gating variable. Hence, we represent the other two variables *v* and *c* as $v = V \, Q_v$ and $c = C \, Q_c$, with dimensionless variables *V* and *C*, respectively. Here, $Q_v$ and $Q_c$ are constants, representing the nominal values of *v* and *c*, respectively. We now derive differential equations for *V* and *C* using that $V' = \frac{1}{Q_v} \, v'$ and $C' = \frac{1}{Q_c} \, c'$. We start with the equation for $V'$:

$$\begin{aligned} V' &{}= \frac{1}{Q_v} \, v' \\ &{}= -\frac{1}{Q_v \, c_m} \, \left( I_{Ca}(v) + I_K(v, n) + I_{K(Ca)}(v, c) \right) \\ &{}= -\frac{1}{Q_v \, c_m} \, \bigg( g_{ca} \, m_{\infty }^2(v) \, (v - e_{ca}) + g_{k}\, n \, (v - e_{k}) + g_{kca} \, \dfrac{c^4}{c^4 + k_s^4} \times (v - e_{k}) \bigg) \\ &{}= -\frac{1}{c_m} \, \bigg( g_{ca} \, m_{\infty }^2(V \, Q_v) \, (V - \bar{e}_{ca}) + g_{k} \, n \, (V - \bar{e}_{k}) + g_{kca} \, \dfrac{C^4}{C^4 + \bar{k}_s^4} \times (V - \bar{e}_{k}) \bigg) , \end{aligned}$$

where $\bar{e}_{ca} = \frac{1}{Q_v} \, e_{ca}$, $\bar{e}_k = \frac{1}{Q_v} \, e_k$, and $\bar{k}_s = \frac{1}{Q_c} \, k_s$. We now define $g_\textrm{max} = \max {(g_{ca}, \, g_{k}, \, g_{kca})}$, so that the rescaled equation for *V* becomes

$$\begin{aligned} V' &{}= -\frac{g_\textrm{max}}{c_m} \, \left( \frac{g_{ca}}{g_\textrm{max}} \, m_{\infty }^2(V \, Q_v) \, (V - \bar{e}_{ca}) + \frac{g_{k}}{g_\textrm{max}} \, n \, (V - \bar{e}_{k}) + \frac{g_{kca}}{g_\textrm{max}} \, \dfrac{C^4}{C^4 + \bar{k}_s^4} \times (V - \bar{e}_{k}) \right) , \end{aligned}$$

which is of the form

$$\begin{aligned} V' = R_v \, f(V, n, C), \end{aligned}$$

with *f* an $\mathcal {O}(1)$ function, because at least one of the ratios $g_{ca}/g_\textrm{max}$, $g_k/g_\textrm{max}$, and $g_{kca}/g_\textrm{max}$ equals 1; indeed, the form of $m_{\infty }(V \, Q_v)$, defined in (4), does not significantly affect the speed associated with the calcium current as it is a function on the unit interval. Hence, $R_v = g_\textrm{max} / c_m$ is the constant that represents the timescale on which *V* evolves. We apply similar steps to the other two equations so that we obtain dimensionless model equations of the form

$$\left\{\begin{aligned} V' &{}= R_v \, f(V, n, C), \\ n' &{}= R_n \, g(V, n, C), \\ C' &{}= R_c \, h(V, n, C), \end{aligned} \right.$$

where the functions *f*, *g* and *h* are all $\mathcal {O}(1)$.

Recall that *n* is already in non-dimensional form and it has timescale constant $R_n = 1/\tau _n$; using the same arguments as for $m_{\infty }(V \, Q_v)$, the expression $n_{\infty }(V \, Q_v)$ (also defined in (4)) has a negligible effect on the order of the right-hand side for *n*.

The non-dimensonalization process for the $C'$-equation is less straightforward. Applying similar steps, however, we find:

$$\begin{aligned} C' &{}= \frac{1}{Q_c} \, c' \\ &{}= -\frac{f_c}{Q_c} \, \left( \alpha \, I_{Ca}(v) + k_p \, c \right) \\ &{}= -\frac{f_c}{Q_c} \, \left( \alpha \, g_{ca} \, m_{\infty }^2(V \, Q_v) \, (V \, Q_v - e_{ca}) + k_p \, C \, Q_c \right) \\ &{}= -\frac{f_c \, \alpha \, Q_v \, g_{ca}}{Q_c} \, \left( m_{\infty }^2(V \, Q_v) \, (V - \bar{e}_{ca})+ \frac{k_p \, Q_c}{\alpha \, Q_v \, g_{ca}} \, C \right) . \end{aligned}$$

The right-hand side of this equation suggests $R_c = f_c \, \alpha \, Q_v \, g_{ca} / Q_c$, but this is only true if the two components in the brackets sum to an $\mathcal {O}(1)$ function. We again use the form of $m_{\infty }(V \, Q_v)$ to claim that the first component is $\mathcal {O}(1)$. Note that the second component is linear in *C* with coefficient $f_c \, k_p / R_c = 0.015 / R_c$ for the default parameter values given in Table 1. Hence, as long as $R_c$ is at least of order $10^{-2}$, this coefficient is at most 1, as required.

During the analysis, we find that $v \in [-65, 10]$ and $c \in [0, 2]$. Therefore, we choose nominal values $Q_v = 100$ and $Q_c= 2$ for the variables *v* and *c*, respectively. The default parameters from Table 1 then give:

16 $$\begin{aligned} \begin{array}{lcccr} R_v =&{} \dfrac{\max {(g_{ca}, \, g_{k}, \, g_{kca})}}{c_m} &{}\approx &{} 716, \\ R_n =&{} \tau _n^{-1} &{}\approx &{} 33, \\ R_c =&{} \dfrac{f_c \, \alpha \, Q_v \, g_{ca}}{Q_c} &{}\approx &{} {1.7}. \end{array} \end{aligned}$$

Note that $R_c$ is sufficiently large to justify the factorization for the $C'$-equation.

### Sodium-potassium minimal model

Using the same calculations as performed for the generic endocrine model above, we obtain the dimensionless sodium-potassium minimal model (5) and (6). Since both *n* and *s* are gating variables in this model, only the equation for *v* needs to be scaled. Again setting $v = V \, Q_v$, we find

$$\left\{\begin{aligned} V' &{}= -\frac{g_\textrm{max}}{c_m} \, \left( \tfrac{g_{L}}{g_\textrm{max}} \, (V - \bar{e}_{L}) + \tfrac{g_{na}}{g_\textrm{max}} \, m_{\infty }(V \, Q_v) \, (V - \bar{e}_{na}) + \tfrac{g_{k}}{g_\textrm{max}} \, n \, (V - \bar{e}_{k}) + \tfrac{g_{km}}{g_\textrm{max}} \, s \, (V - \bar{e}_{k}) - \frac{1}{g_\textrm{max} \, Q_v} \, I \right) , \\ n' &{}= \frac{1}{\tau _n} \, \left( n_{\infty }(V \, Q_v) - n \right) , \\ s' &{}= \frac{1}{\tau _s} \, \left( s_{\infty }(V \, Q_v) - s \right) , \end{aligned} \right.$$

where $\bar{e}_{L} = \frac{1}{Q_v} \, e_L$, $\bar{e}_{na} = \frac{1}{Q_v} \, e_{na}$, $\bar{e}_{k} = \frac{1}{Q_v} \, e_k$, and $g_\textrm{max} = \max {(g_L, g_{na}, \, g_{k}, \, g_{km})}$. Note that the actual timescale constants for the sodium-potassium minimal model do not depend on the chosen value for $Q_v$; indeed, $m_{\infty }(V \, Q_v)$ as defined in (6) has no significant effect for the same reasons as in the generic endocrine model, and we can assume $Q_v$ is chosen such that $1 / (g_\textrm{max} \, Q_v) \le 1$. By setting all parameters to their default values given in Table 2, we find timescale constants

$$\begin{aligned} \begin{array}{lcccr@{.}l} R_v =&{} \dfrac{\max {(g_L, g_{na}, \, g_{k}, \, g_{km})}}{c_m} &{}\approx &{} 20.0, \\ R_n =&{} \tau _n^{-1} &{}\approx &{} 6.6, \\ R_s =&{} \tau _s^{-1} &{}\approx &{} 0.005, \end{array} \end{aligned}$$

which represent the relative speeds of *v*, *n* and *s*, respectively.

### Minimal Chay–Keizer model

The equations in terms of dimensionless variables for the minimal Chay–Keizer model (7) and (8) are derived in complete analogy to the other derivations. The only difference is that the timescale constant in the equation for the gating variable *n* is now a function of *v*, denoted $\tau _n(v)$. Since $v \in [-65, 0]$ and $c \in [0, 6]$ , we choose nominal values $Q_v = 100$ for $V = \frac{1}{Q_v} \, v$ and $Q_{c}= 6$ for $C = \frac{1}{Q_{c}} \, c$. Observing from the analysis that $\tau _n(v) = \tau _n(V \, Q_v) \in [10,\, 20]$, we find for the default parameters given in Table 3:

$$\begin{aligned} \begin{array}{lcccl} R_v =&{} \dfrac{\max {(g_{ca}, \, g_{k}, \, g_{kca})}}{c_m} &{}\approx &{} 1.8, \\ R_n =&{} \dfrac{1}{\tau _n(V \, Q_v)} &{}\in &{} [0.05,\, 0.1], \\ R_c =&{} \dfrac{f_c \, \alpha \, Q_v \, g_{ca}}{Q_{c}} &{}\approx &{} 0.004. \end{array} \end{aligned}$$

Note that the timescale constant $R_c$ for the slow variable *c* requires $f_c \, k_p / R_c \le 1$, but with $f_c = 0.0058$ and $k_p = 0.00513 \, \textrm{ms}^{-1}$ in Table 3, this is certainly satisfied if $R_c$ is of order $10^{-3}$.

### Butera model

Again in complete analogy to the earlier models, we find timescale constants representing the relative speeds for the variables *v*, *n*, and *p* in the Butera model (9) and (10). Based on the default parameter values given in Table 4, we find timescale constants:

$$\begin{aligned} \begin{array}{lcccl} R_v =&{} \dfrac{\max {(g_L,\, g_{na}, \, g_{k}, \, g_{nap}, \, g_{ton})}}{c_m} &{}\approx &{} 1.33, \\ R_n =&{} \tau _n^{-1} &{}\approx &{} 0.17, \\ R_p =&{} \dfrac{1}{\tau _p(V \, Q_v)} &{}\in &{} [10^{-4}, \, 10^{-3}]. \end{array} \end{aligned}$$
