# Supplementary figures and images for: Slow negative feedback enhances robustness of square-wave bursting
Source: J Comput Neurosci. 2023 Apr 17;51(2):239–61. doi: 10.1007/s10827-023-00846-y (PMC10181982; doi:10.1007/s10827-023-00846-y)

**(A)**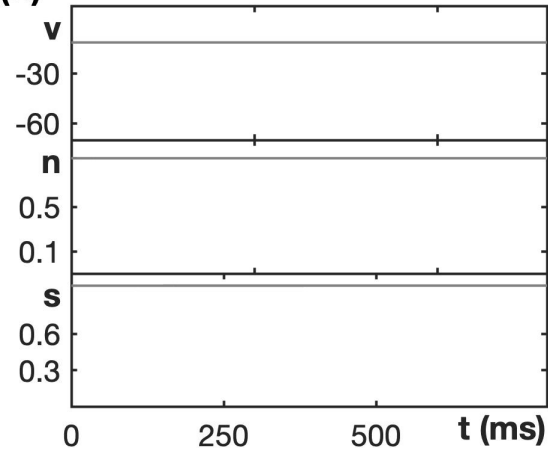**(B)**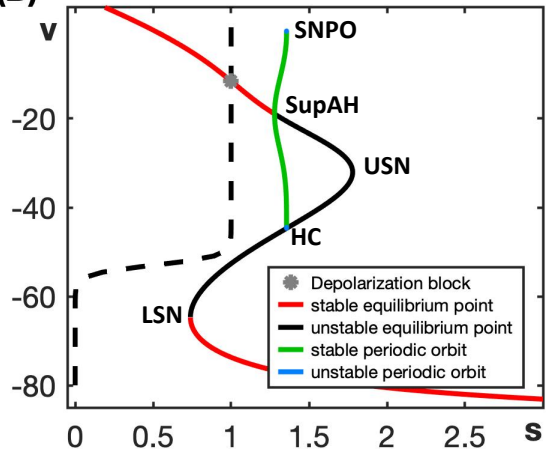

Supplement: Supplementary file 1 — A Depolarization block exhibited by the sodium-potassium minimal model (5)-(6) resulting from a full-system stable steady state $(v, n, s) \approx (-11.5,0.93,1.0)$(v,n,s)≈(-11.5,0.93,1.0) at elevated voltage for $g_{na} = 35$gna=35. B Bifurcation diagram of the model's fast system associated with (A). The stable steady state lies where the fast subsystem equilibrium curve intersects the s-nullcline (dashed black) (pdf 121 KB) [file 10827_2023_846_MOESM1_ESM.pdf]

**(A)**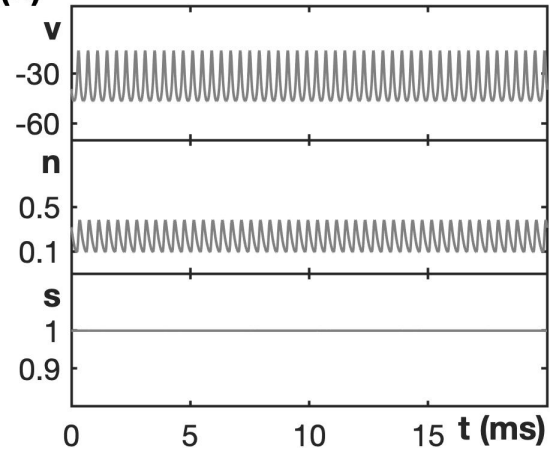**(B)**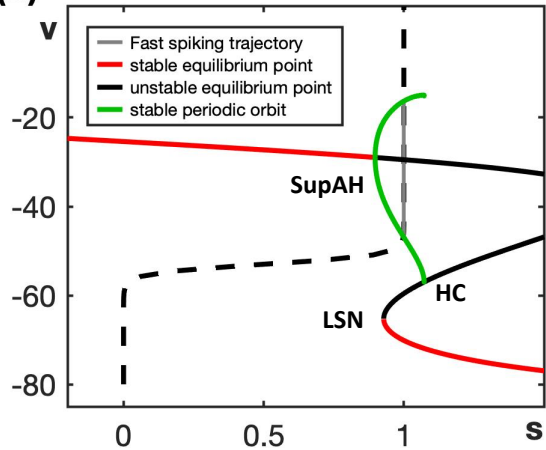

Supplement: Supplementary file 2 — A Fast spiking exhibited by the modified sodium-potassium minimal model (5)-(6), (12)-(13) resulting from a full-system stable periodic orbit with $s \approx 1.0$s≈1.0 for $g_{na} = 39$gna=39 and $1/\tau_h=13$1/τh=13. B Bifurcation diagram of the model's fast system associated with (A) (pdf 258 KB) [file 10827_2023_846_MOESM2_ESM.pdf]

**(A)**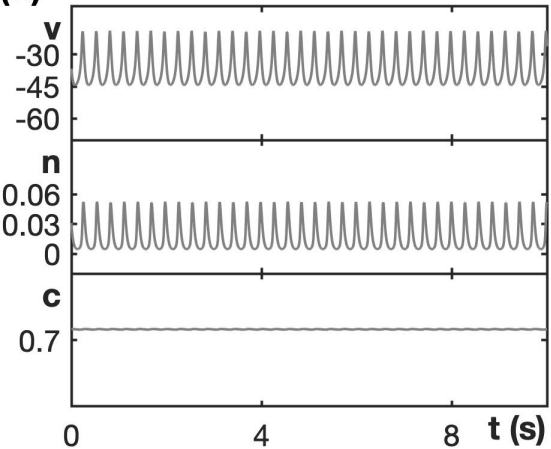**(B)**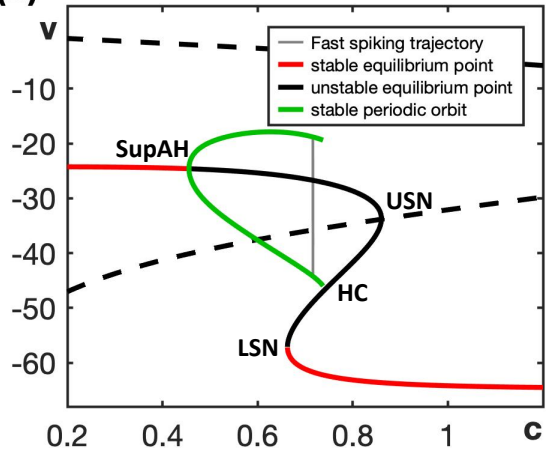

Supplement: Supplementary file 3 — A Fast spiking exhibited by the generic endocrine model (3)-(4) resulting from a full-system stable periodic orbit with $c \approx 0.7$c≈0.7 for $g_{ca} = 0.81$gca=0.81 and $\alpha=2$α=2. B Bifurcation diagram of the model's fast system associated with (A) (pdf 290 KB) [file 10827_2023_846_MOESM3_ESM.pdf]

(A)

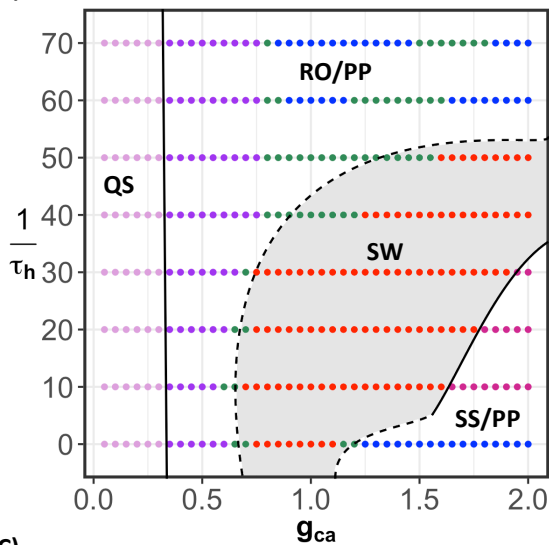

(B)

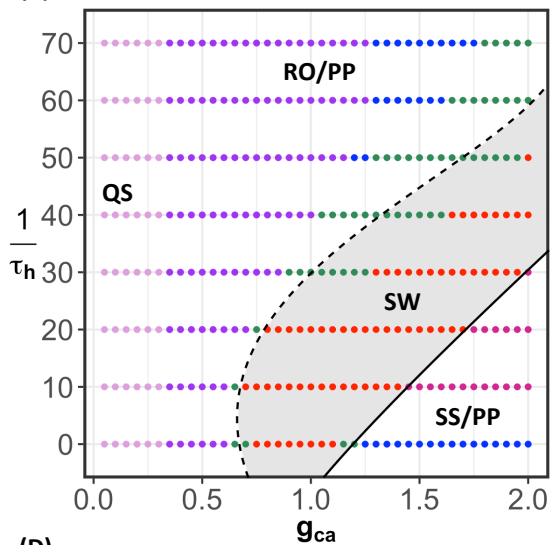

(C)

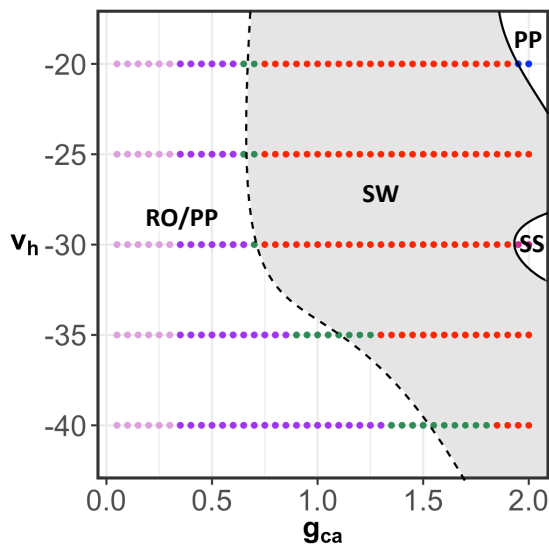

(D)

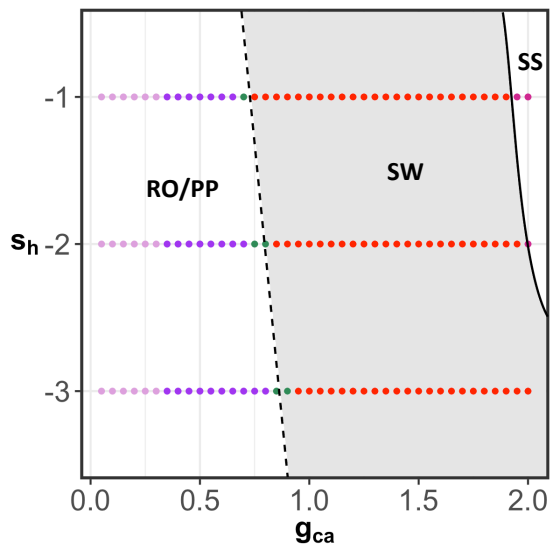

Supplement: Supplementary file 4 — Two-parameter bifurcation diagrams of the modified generic endocrine model (3)-(4), (11), and (13), with respect to A $g_{ca}$gca and $1/\tau_h$1/τh for $v_h=-30$vh=-30 and $s_h=-1$sh=-1; B $g_{ca}$gca and $1/\tau_h$1/τh for $v_h=-35$vh=-35 and [file 10827_2023_846_MOESM4_ESM.pdf]
